# Neutrophil extracellular traps potentiate effector T cells via endothelial senescence in uveitis

**DOI:** 10.1172/jci.insight.180248

**Published:** 2025-01-23

**Authors:** Zuoyi Li, Zhuang Li, Yunwei Hu, Yanyan Xie, Yuxun Shi, Guanyu Chen, Jun Huang, Zhiqiang Xiao, Wenjie Zhu, Haixiang Huang, Minzhen Wang, Jianping Chen, Xiaoqing Chen, Dan Liang

**Affiliations:** 1 State Key Laboratory of Ophthalmology, Zhongshan Ophthalmic Center, Sun Yat-sen University, Guangdong Provincial Key Laboratory of Ophthalmology Visual Science, Guangzhou, China.; 2 Ophthalmic Center, The Second Affiliated Hospital, Jiangxi Medical College, Nanchang University, Nanchang, China.

**Keywords:** Autoimmunity, Ophthalmology, Autoimmune diseases, Cellular immune response, Neutrophils

## Abstract

Autoimmune uveitis (AU) is a sight-threatening ocular autoimmune disorder that often manifests as retinal vasculitis. Increased neutrophil infiltration around retinal vessels has been reported during the progression of AU, while how they function is not fully recognized. Neutrophil extracellular traps (NETs), produced by activated neutrophils, have been suggested to be detrimental in autoimmune diseases. Here, we found that NETs were elevated in patients with active AU, and this was verified in an experimental AU (EAU) mouse model. Depletion of neutrophils or degradation of NETs with deoxyribonuclease-I (DNase I) could decrease CD4^+^ effector T cell (Teff) infiltration in retina and spleen to alleviate EAU. Moreover, we found that the expression of adhesion molecules, selectin, and antigen-presenting molecules was elevated in EAU retina and in retinal microvascular endothelial cells (RMECs) cocultured with NETs. The stimulated RMECs further facilitated CD4^+^ T cell adhesion, activation, and differentiation into Teffs. Mechanistically, NETs trigger RMEC activation by hastening cell senescence through the cyclic GMP-AMP synthase (cGAS)/stimulator of interferon genes (STING) pathway. Slowing down senescence or inhibiting the cGAS/STING pathway in RMECs reduces the activation and differentiation of CD4^+^ T cells. These results suggest a deleterious role of NETs in AU. Targeting NETs would offer an effective therapeutic method.

## Abstract 2

Neutrophil extracellular traps potentiate effector T cells through inducing retinal microvascular endothelial cell senescence to contribute to autoimmune uveitis development.

## Introduction

Autoimmune uveitis (AU) is an ocular inflammatory disease that predominantly affects working age people and is among the leading causes of vision loss; it is characterized by inflammation of the uvea and retina (1, 2). Retinal vasculitis is a common blinding clinical manifestation of AU, during which activated immune cells flock into the retina through the blood-retinal barrier (BRB), resulting in irreversible retinal structural destruction and functional impairment (3–5). At the peak of experimental AU (EAU), a murine model that manifests AU well, simultaneous surges of neutrophils and CD4^+^ T cells are found in the retina (6). Nowadays, it’s generally accepted that AU is mainly driven by activated CD4^+^ effector T cells (Teffs) (2, 5); whereas, how neutrophil function in AU requires further investigation.

Neutrophils are considered as the primary defense in innate immunity against microbial organisms (7). They are the spearhead that arrives at inflammatory sites and maintains inflammation (8). When neutrophils are activated by stimuli such as complements, cytokines, autoantibodies, or immune complexes, they can release nuclear and granular components, which arrange themselves into broad, web-like formations of DNA, called neutrophil extracellular traps (NETs) (9, 10). Aside from bacteria capturing ability during infection, NETs exert a pivotal pathogenic role in autoimmune disorders, such as systemic lupus erythematosus (SLE), rheumatoid arthritis (RA), and inflammatory bowel disease (IBD), through damaging host cells directly, presenting autoantigens or activating immune cells (10–12). Moreover, NETs have been verified to induce Th17 differentiation in psoriasis and asthma (13, 14) in addition to inducing Th1 polarization via dendritic cell activation in type 1 diabetes (15), suggesting that NETs regulate Teffs in these autoimmune diseases. However, whether NETs exert their function through regulating T cells in AU has yet to be unveiled.

Endothelial cells (ECs) maintain the integrity of the blood vessel barrier, which constitutes the innate immune system and maintains immune homeostasis (16). Under pathogenic circumstances, ECs become activated, manifesting greater vascular permeability, secreting proinflammatory cytokines and chemokines, and expressing adhesion molecules and antigen-presenting molecules (17, 18). This phenotypic change in ECs enables them to promote immune cell trafficking, activation, and function, including that in T cells (16, 19). In addition, excessive NET release influences ECs function to regulate progression of disease, including SLE and IBD (12, 20). Thus, we wondered whether ECs are involved in the regulation of CD4^+^ T cells by NETs.

Therefore, we aimed to study the influence of NETs on AU and associated pathogenesis. We investigated the concentration of NETs between individuals with AU and healthy individuals and the effect of NETs on retinal EC function as well as subsequent CD4^+^ T cell activation and differentiation.

## Results

### Elevated NETs were detected in both patients with active AU and EAU mice.

**Figure 1:**
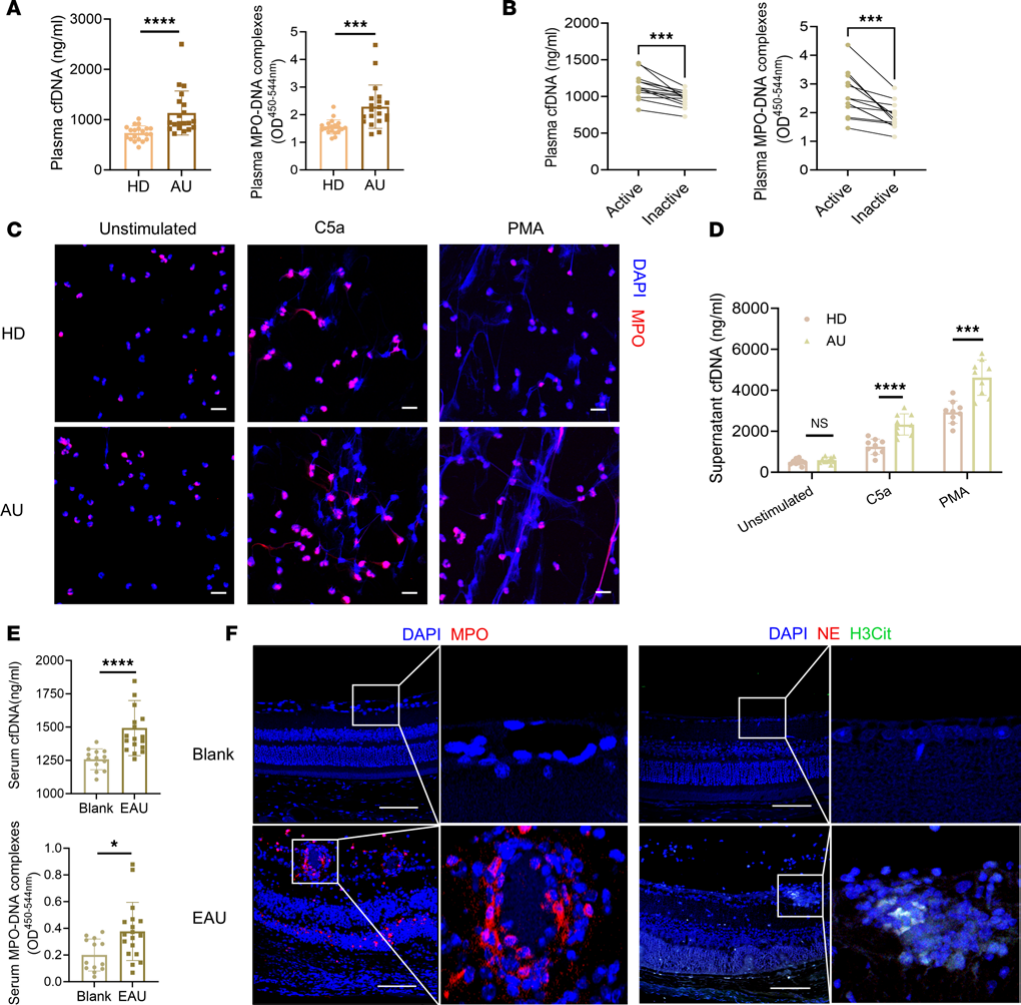
NET formation was increased in patients with active AU and in the EAU mouse model. (**A**) cfDNA and the MPO-DNA complex were detected in the plasma of patients with active AU and healthy donors (*n* = 20). (**B**) Longitudinal comparison of cfDNA and MPO-DNA in plasma of patients with AU in both the active phase and remission/drug-inactive phase (*n* = 13). (**C**) Human neutrophils were isolated and stimulated with PBS, complement C5a, or PMA, and representative immunofluorescence staining showed neutrophils and NETs costained with MPO (red) and DAPI (blue). Scale bar: 20 μm. (**D**) The cell supernatants from 3 groups were detected for cfDNA concentration (*n* = 9). (**E**) Sera from EAU (*n* = 18) and blank (*n* = 13) mice were also collected for cfDNA and MPO-DNA complex detection. (**F**) Mouse eyeballs were enucleated for paraffin sectioning and immunofluorescence, and representative images of immunofluorescence staining of neutrophils and NETs in retinas staining MPO (red) and DAPI (blue) or costaining for NE (red), H3Cit (green) and DAPI (blue) are shown. Scale bar: 50 μm. Representative data are from at least 3 independent experiments. Data are presented as the mean ± SD. *P < 0.05, **P < 0.01, ***P < 0.001, ****P < 0.0001 for Mann-Whitney test (**A**, **D**, and **E**) and nonparametric paired *t* test (**B**).

To verify the level of circulating NETs in patients with AU and healthy donors (HDs), we conducted an examination of cell-free DNA (cfDNA) and myeloperoxidase-DNA (MPO-DNA) complex (21), which are representative markers of NETs in circulation. The plasma cfDNA level was higher in patients with active AU than in HDs (Figure 1A). The expression of MPO-DNA complex was also notably elevated in patients with active AU (Figure 1A). All patients included in this assay were diagnosed with active noninfectious panuveitis (including idiopathic uveitis and Behcet’s uveitis), presenting with prominent retinal vasculitis on fundus fluorescein angiography. We compared the plasma NETs levels between patients with active idiopathic uveitis without systemic involvement and patients with Behcet’s uveitis and found no significant difference (Supplemental Figure 1; supplemental material available online with this article; https://doi.org/10.1172/jci.insight.180248DS1). This result suggests that NETs levels are not closely associated with systemic involvement. Additionally, plasma samples were collected from patients during their active phase and remission phase or inactive phase with treatment. A longitudinal comparison revealed that NETs levels were significantly lower when disease was inactive (Figure 1B), indicating the role of NETs in disease activation.

Moreover, fresh neutrophils from patients with active AU and HDs were isolated so they remained unstimulated or stimulated with complement C5a or PMA, which are both common activator of NET production (21–23). The neutrophils were stained with MPO, a biomarker of neutrophil activation and one of the main components of NETs (21). More fibrous and web-like NETs were observed in stimulated neutrophils from patients with AU than from HDs, with PMA being stronger than C5a, while the unstimulated neutrophils from both groups remained almost unchanged in immunofluorescence (Figure 1C). We also collected the cell supernatants and measured the cfDNA concentration. The unstimulated neutrophils from patients with AU produced almost the same mounts of NETs as those from HDs. After stimulation, NET production increased significantly in the active uveitis group compared with that in the control group (Figure 1D). The in vitro NET release assay showed that neutrophils from patients with active AU were more susceptible to producing NETs than those from healthy individuals when stimulated.

To verify our clinical findings, we further tested NETs in the sera of EAU mice and healthy controls. As expected, significantly elevated cfDNA and MPO-DNA levels were detected in EAU (Figure 1E). Then, we performed retinal immunofluorescence staining of key proteins for NET production, including MPO, neutrophil elastase (NE), and citrullinated histone H3 (H3Cit), to determine whether NETs could form locally (24, 25). Our results showed distinct staining was scattered in EAU mouse retina, especially around retinal vessels and in the retinal granuloma and retinal folds (Figure 1F). However, there was almost no staining in blank mouse retina. The elevated staining indicated neutrophil infiltration and concomitant increased NET production in the EAU disease process. As deoxyribonuclease I (DNase I) is responsible for NET degradation in vivo (26–28), we further measured DNase I concentration and activity in the plasma of patients with AU and healthy controls as well as in the serum of EAU mice and normal mice. The results showed no significant difference between the AU and healthy control groups in either humans or mice, indicating that NET degeneration wasn’t impaired in uveitis (Supplemental Figure 2, A and B). To conclude, we demonstrated that NET release was augmented in patients with active AU and EAU mice.

### Neutrophil depletion and/or NET degradation alleviated the severity of EAU.

**Figure 2:**
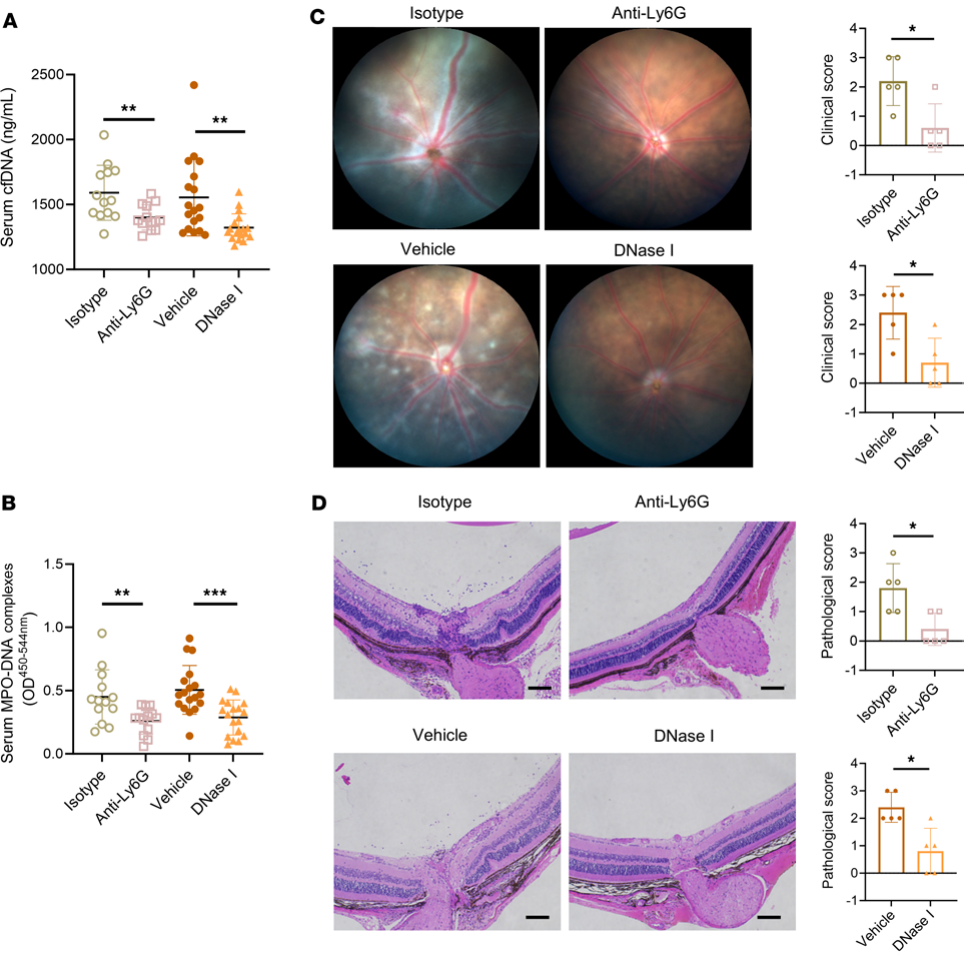
NET degradation downregulated circulating NETs and alleviated retina vasculitis severity of EAU. (**A** and **B**) Fourteen days after immunization, the severity of EAU peaked. Sera from EAU mice treated with isotype control (*n* = 13), anti-Ly6G antibody (*n* = 13), vehicle (*n* = 18), or DNase I (*n* = 18) were collected to detect cfDNA and MPO-DNA complex levels. (**C**) Fundus photography of EAU mice treated with anti-Ly6G antibody, DNase I, as well as control vehicle was taken, and the corresponding clinical score statistical chart is shown (*n* = 5). (**D**) H&E staining was also performed on day14 to assess the histopathological score (scale bar: 200 μm) (*n* = 5). Representative data are from at least 3 independent experiments. Data are presented as the mean ± SD. *P < 0.05, **P < 0.01, ***P < 0.001 for Mann-Whitney test.

To further investigate the role of NETs in the pathogenesis of EAU, we treated EAU mice with anti-Ly6G antibody or DNase I, which could eliminate neutrophils or degrade NETs, respectively (12, 24, 29). Notably, both cfDNA and the MPO-DNA complex were decreased by either anti-Ly6G antibody or DNase I treatment. These findings indicated that circulating NETs might be mostly derived from neutrophils (Figure 2, A and B). Representative images of the EAU fundus were taken on day 14 after modeling to evaluate the disease severity. The retinas of EAU mice administered with PBS showed severe uveitis characterized by optic disk edema, diffuse inflammatory lesions, and extensive vasculitis. However, EAU mice treated with anti-Ly6G antibody or DNase I exhibited an almost normal retina or slight vasculitis (Figure 2C). The eyeballs were also analyzed pathologically; the retina folds, granulomatous lesions, and inflammatory cell infiltration were decreased after drug interruption (Figure 2D). Taken together, these results revealed that neutrophils participate in the pathogenesis of EAU through NETs, blocking them could reduce the disease severity.

### Neutrophil depletion or NET degradation modulated the intraocular inflammatory profile in EAU.

**Figure 3:**
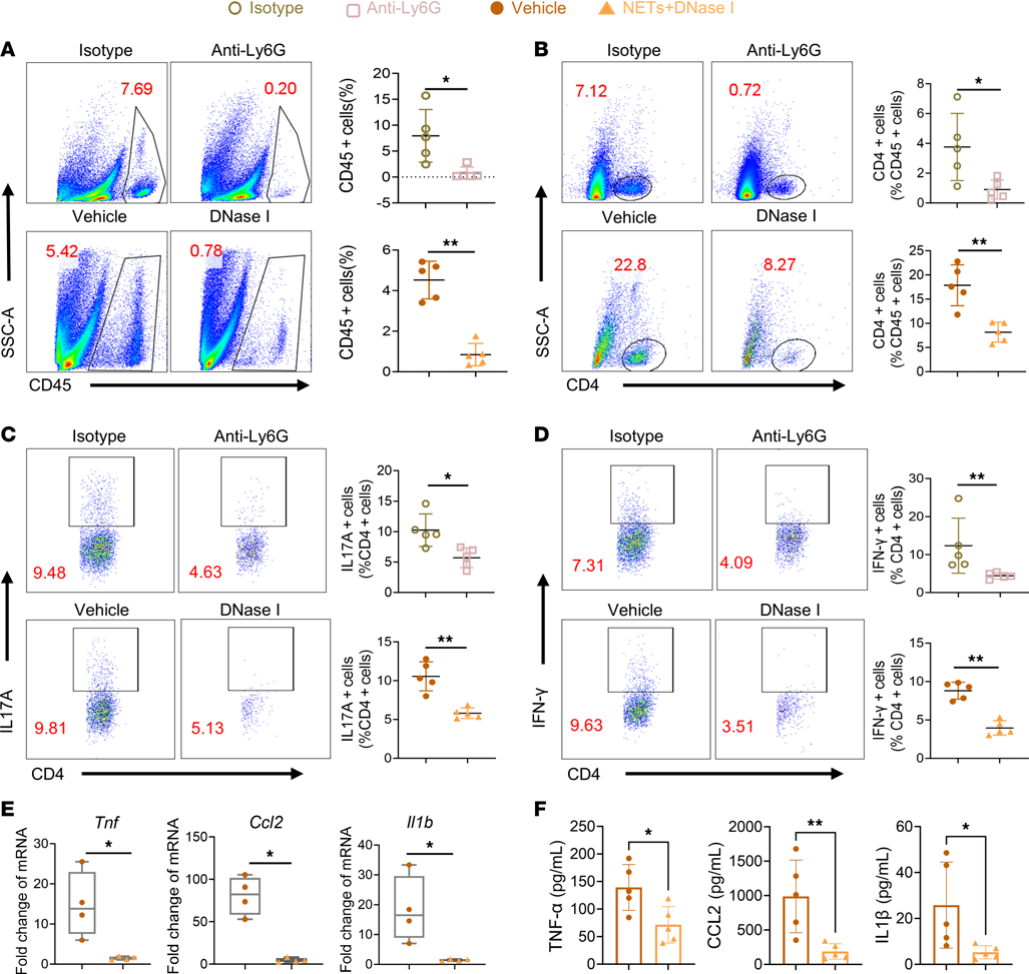
Neutrophil depletion or DNase I treatment could reduce retinal inflammatory infiltration in EAU. (**A**–**D**) Intraocular lymphocytes (CD45^+^), CD4^+^ T cells (CD45^+^CD4^+^), Th17 cells (CD45^+^CD4^+^IL-17A^+^), and Th1 cells (CD45^+^CD4^+^IFN-γ^+^) from EAU mice treated with anti-Ly6G antibody, DNase I, or control vehicle were assayed and evaluated with flow cytometry 14 days after immunization (*n* = 5). (**E**) The mRNA expression levels of TNF-α, CCL2, and IL-1β in the retinas were detected via RT-qPCR (*n* = 4). (**F**) The protein expression levels of TNF-α, CCL2, and IL-1β in the retinas were detected via ELISA (*n* = 5). Representative data are from at least 3 independent experiments. Data are presented as the mean ± SD. *P < 0.05, **P < 0.01 for Mann-Whitney test.

Active lymphocyte infiltration into the eyes is the most important pathological process in EAU (30); therefore, we digested the retina for immune cell analysis 14 days after immunization. The results showed that, compared with control treatment, DNase I or anti-Ly6G treatment decreased intraocular leukocyte accumulation (Figure 3A). As the key pathogenesis of uveitis is the immunoinflammatory response mediated by CD4^+^ T cells (2), we further analyzed the infiltrating cells and found that the frequency of intraocular CD4^+^ T cells was decreased by DNase I or anti-Ly6G treatment (Figure 3B). Among CD4^+^ T cells, Th17 and Th1 cells are essential effector cells marked by secreting inflammatory cytokine IL-17A and IFN-γ, respectively (31). We found that the Th17 and Th1 frequencies were also reduced after treatment (Figure 3, C and D). Moreover, we applied real-time quantitative PCR (RT-qPCR) and ELISA to investigate the gene and protein expression of proinflammatory cytokines. The results showed significantly decreased expression of TNF-α (*Tnf*), CCL2 (*Ccl2*) and IL-1β (*Il1b*) under DNase I treatment (Figure 3, E and F). Taken together, these findings suggested that anti-Ly6G or DNase I could alleviate uveitis by reducing inflammatory cell infiltration and cytokine expression in the retina.

### Neutrophil depletion or NET degradation attenuated systemic inflammation in EAU.

**Figure 4:**
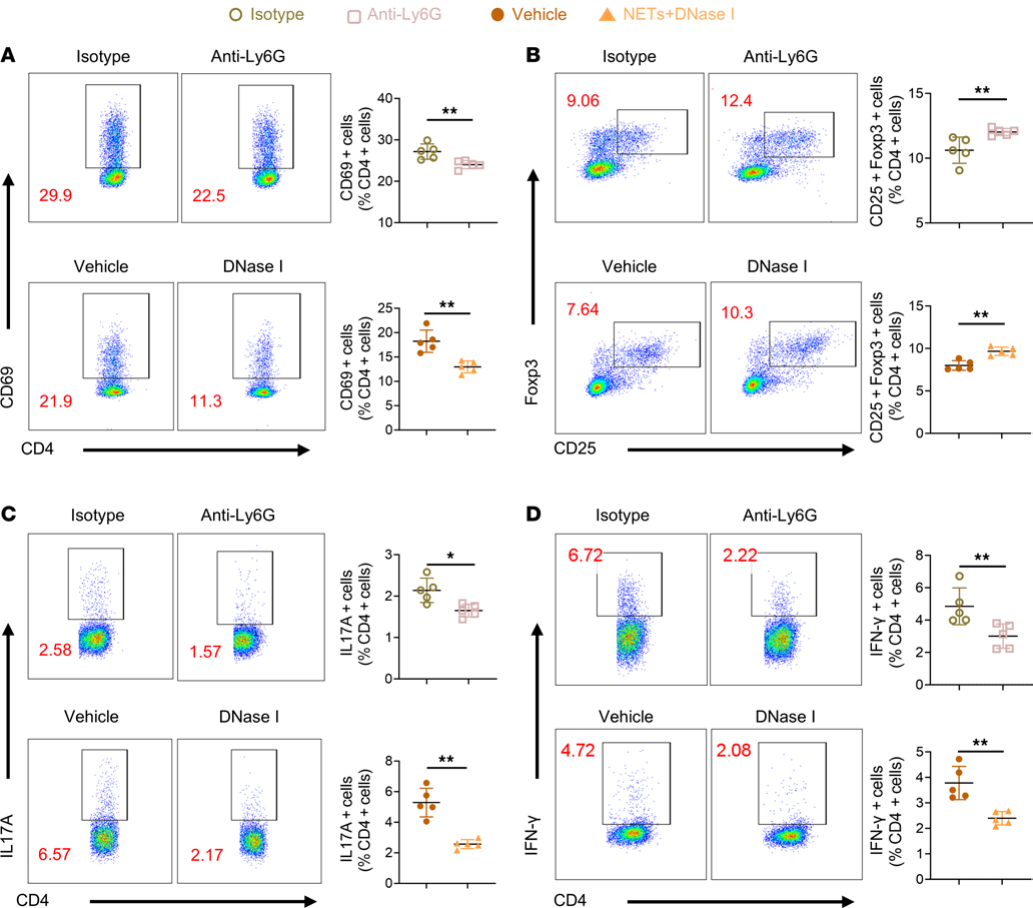
Anti-Ly6G antibody and DNase I modulated systemic immune profiles. (**A**–**D**) Lymphocytes from the spleen were assayed through flow cytometry to evaluate the ratio of early activated (**A**) CD4^+^ T cells (CD4^+^CD69^+^), (**B**) Tregs (CD4^+^CD25^+^Foxp3^+^), (**C**) Th17 cells (CD4^+^IL-17A^+^), and (**D**) Th1 cells (CD4^+^IFN-γ^+^) from EAU mice treated with anti-Ly6G antibody, DNase I, or control vehicle on day 14 after immunization (*n* = 5). Representative data are from at least 3 independent experiments. Data are presented as the mean ± SD. *P < 0.05, **P < 0.01 for Mann-Whitney test.

The spleen, the center of cellular and humoral immunity, is the largest immune organ. When the BRB is disrupted, proinflammatory immune cells in the immune organs circulate into the retina to cause AU (3). Therefore, we assayed activated T cells, Teffs, and Tregs in the spleen to evaluate the therapeutic efficacy of DNase I or neutrophil depletion. CD69 is one of the earliest molecules expressed on T cells when activated (32). The percentage and absolute number of CD69^+^CD4^+^ T cells was decreased in the presence of anti-Ly6G or DNase I (Figure 4A and Supplemental Figure 3A). Tregs can suppress immune responses (33), and DNase I or anti-Ly6G increased Tregs compared with control (Figure 4B and Supplemental Figure 3B). A reduced frequency and number of Th17 and Th1 cells were also detected in the treatment group (Figure 4, C and D, and Supplemental Figure 3, C and D). Above all, DNase I treatment or neutrophil depletion deterred the progression of EAU by inhibiting the CD4^+^ T cells activation and restoring the Teff/Treg balance.

### NETs activated ECs.

**Figure 5:**
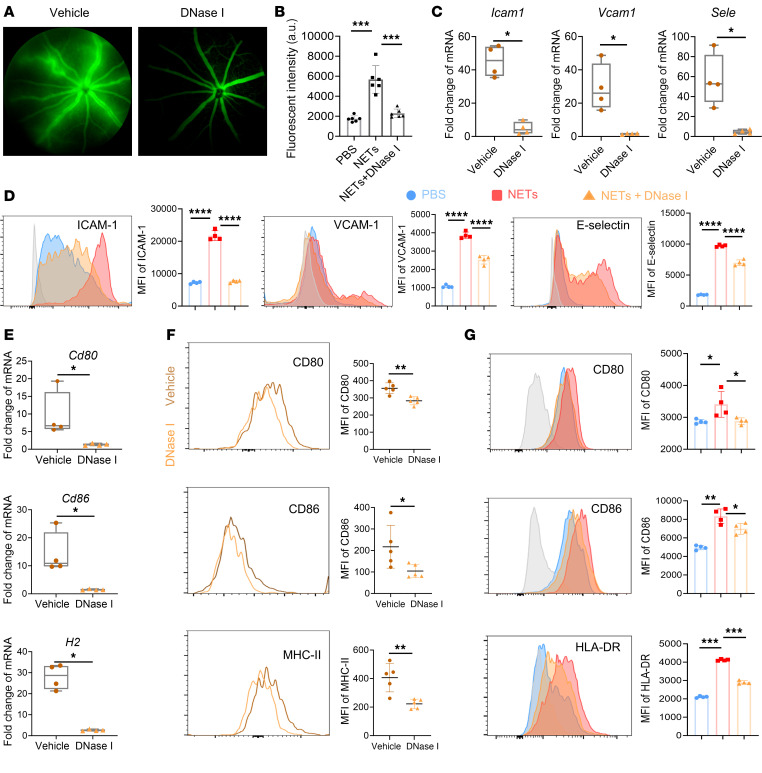
NETs activated RMECs. (**A**) Fluorescein sodium was injected intraperitoneally to evaluate retinal vascular leakage on day14, and the representative fundus images are shown. (**B**) RMECs were cultured in the upper chamber of the Transwell assay and treated with PBS, NETs or NETs pretreated by DNase I for 24 hours. The fluorescence intensity of FITC-dextran that passed through RMECs to the lower chamber was measured (*n* = 6). (**C**) The mRNA expression levels of ICAM-1, VCAM-1, and E-selectin in retinas from blank, EAU, and DNase I–treated EAU mice were detected via RT-qPCR (*n* = 4). (**D**) The protein expression of the above molecules in RMECs was determined by flow cytometry after coincubation with PBS, NETs, or NETs pretreated by DNase I (*n* = 4). (**E**) The mRNA expression levels of antigen-presenting molecules in mouse retinas were detected via RT-qPCR (*n* = 4). (**F**) The expression of antigen-presenting molecules (CD80, CD86, and HLA-DR) on retinal endothelial cells from mice treated differently were detected by flow cytometry (*n* = 5). (**G**) The expression of the antigen-presenting molecules in RMECs after in vitro stimulation was determined by flow cytometry (*n* = 4). Representative data are from at least 3 independent experiments. Data are presented as the mean ± SD. *P < 0.05, **P < 0.01, ***P < 0.001, ****P < 0.0001 for Mann-Whitney test (**C**, **E**, and **F**) and 1-way ANOVA (**B**, **D**, and **G**).

BRB plays a pivotal role in maintaining retinal homeostasis. Breakdown of the barrier leads to the entry of inflammatory cells into the eyeball, resulting in structural damage and impaired function (5). In our study, fluorescein fundus angiography revealed obvious dye leakage in blood vessels, with significant inflammation in EAU retina, which indicated BRB disruption. We treated EAU mice with DNase I and found obvious alleviation of vascular leakage (Figure 5A). Next, we examined whether NETs could impair BRB integrity in vitro. We carried out endothelial permeability assay to evaluate vascular barrier function (34), and found increased EC permeability of FITC-dextran after NETs stimulation (Figure 5B). These data indicated that NETs were responsible for BRB disruption in EAU.

Retinal microvascular ECs (RMECs) constitute the main component of the BRB; therefore, we investigated their functional changes in EAU. Adhesion molecules and selectin, such as ICAM-1, VCAM-1, and E-selectin, are molecules on ECs that influence BRB integrity as well as mediate adhesion with lymphocytes and the migration of lymphocytes from endothelium into the tissue (35). Thus, we isolated mouse retinas for RT-qPCR analysis and found that the mRNA expression of endothelial activation–related molecules ICAM-1 (*Icam1*), VCAM-1 (*Vcam1*), and E-selectin (*Sele*) was significantly greater in EAU mice than in DNase I–treated mice (Figure 5C). In vitro, the protein expression of these genes in RMECs was upregulated by NETs and downregulated by DNase I, as determined by flow cytometry analysis (Figure 5D). To conclude, we demonstrated that NETs induce an inflammatory phenotype in ECs in EAU.

MHC-II comprises a group of glycoproteins expressed on antigen-presenting cells that activate CD4^+^ T cells. CD80 and CD86 are costimulatory molecules that assist in antigen presentation. Several studies have demonstrated that ECs can convert into antigen-presenting cells after cytokine stimulation (16), and we wondered if NETs could elevate the expression of these molecules on ECs in EAU. Thus, we analyzed expression of antigen-presenting related molecules CD80 (*Cd80*), CD86 (*Cd86*), and MHC-II (*H2*) in mouse retina with RT-qPCR assay and found they were significantly reduced with DNase I treatment, which was confirmed by flow cytometry analysis of mouse retinal ECs (Figure 5, E and F). In vitro, we detected that NETs increased the expression of the above molecules on RMECs, and these increases were partly reversed by DNase I (Figure 5G). These data suggested that NETs could turn RMECs into antigen-presenting cells in EAU. Together, these findings reveal that NETs activate RMECs and possibly influence CD4^+^ T cells in AU.

### NETs regulated CD4^+^ T cells through RMECs.

**Figure 6:**
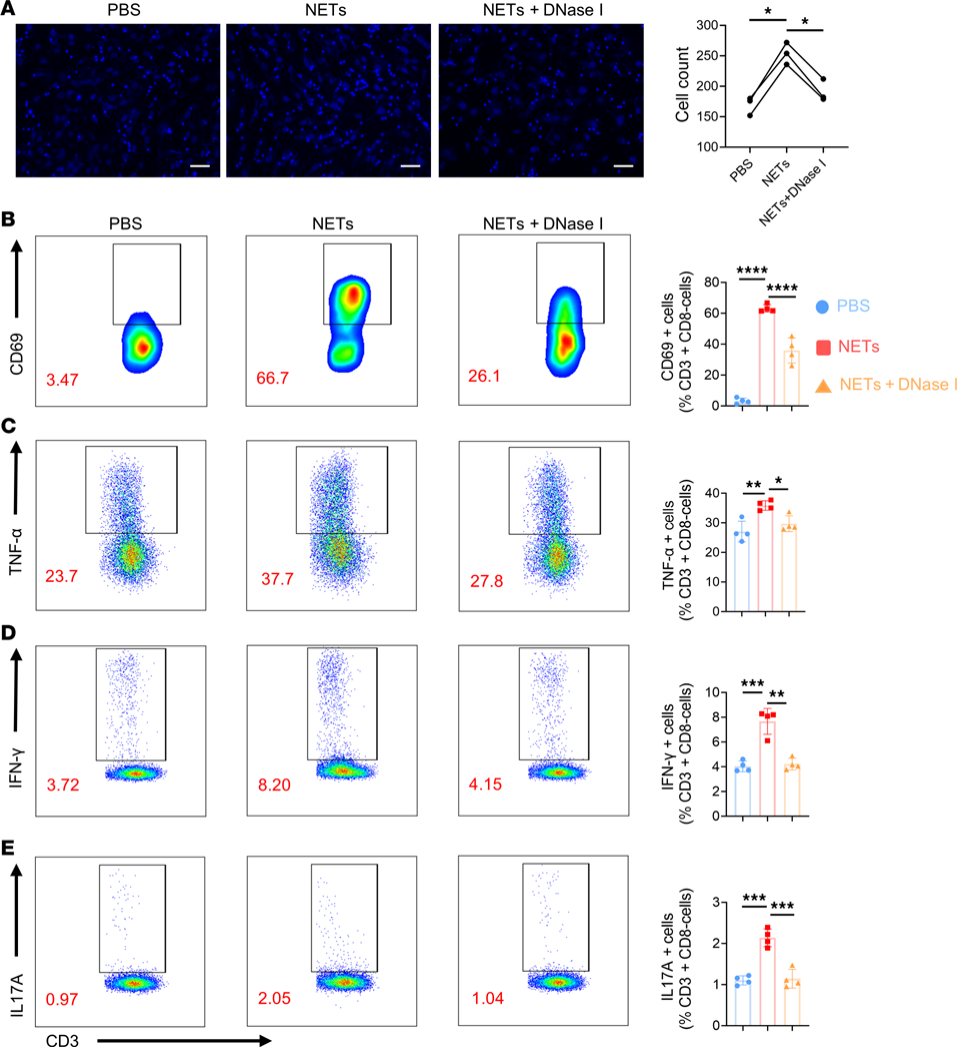
RMECs stimulated by NETs induced CD4^+^ T cell adhesion, activation, and differentiation. (**A**) Immunofluorescence of CD4^+^ T cells attached to RMECs after stimulation by PBS, NETs, or NETs pretreated with DNase I (*n* = 3). Scale bar: 100 μm. (**B**) CD4^+^ T cells cocultured with NETs for 6 hours were collected and stained for early activated CD4^+^T cells (CD3^+^CD8^–^CD69^+^) via flow cytometry (*n* = 4). (**C**–**E**) After coculture for 24 hours, all the T cells were collected and stained for TNF-α, IFN-γ, and IL-17A via flow cytometry (*n* = 4). Statistical graphs of the proportion of CD69^+^CD4^+^ T cells, Th17 cells, and Th1 cells are shown. Representative data are from at least 3 independent experiments. Data are presented as the mean ± SD. *P < 0.05, **P < 0.01, ***P < 0.001, ****P < 0.0001 for 1-way ANOVA.

As indicated in the above results, NETs induced RMECs to exhibit increased expression of adhesion molecules, selectin, and antigen-presenting related molecules, which are relevant to lymphocyte activation and adhesion. Thus, we conducted additional experiments to further examine the changes in CD4^+^ T cell function, after coculture with RMECs that were activated by NETs. We first stimulated RMECs with PBS, NETs, or NETs pretreated with DNase I for 24 hours; then cocultured RMECs with isolated human CD4^+^ T cells; and stained with DAPI. The nuclei of T cells were small, bright, and round, while the nuclei of RMECs were large, dim, and elliptical. Cell adhesion is an essential step for lymphocytes to travel through the vascular wall into tissue (19). RMECs activated with NETs showed significant elevated adhesion to CD4^+^ T cells, while the addition of DNase I lowered the adhered cell count (Figure 6A). We also performed flow cytometry to further investigate the phenotype changes in the T cells. NET-treated RMECs activated peripheral CD4^+^ T cells from patients with AU at a markedly greater ratio than did DNase I–treated RMECs (Figure 6B). Importantly, the proportions of Teffs expressing TNF-α, IFN-γ, and IL-17A also showed a consistent trend (Figure 6, C–E). Thus, the results showed that the RMECs, which were stimulated with NETs, could promote CD4^+^ T cell adherence, activation, and differentiation into Teffs (Th1 and Th17), while DNase I preferentially blocked this conversion.

### NET regulated RMECs function by senescence through the cGAS/STING pathway.

**Figure 7:**
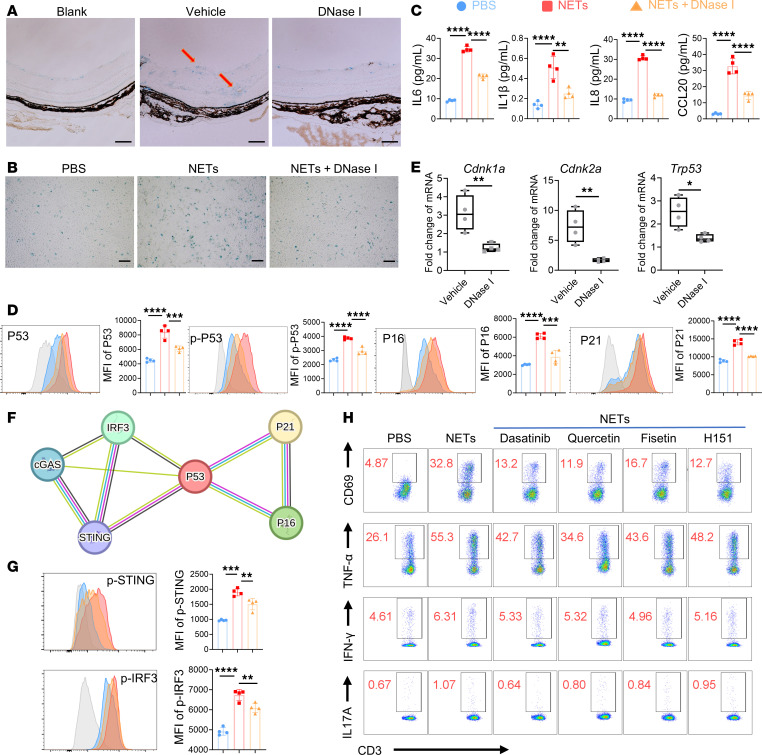
NETs induced RMEC senescence through the cGAS/STING pathway. (**A**) Representative images of SA-β-gal–stained retina sections from blank mice, and EAU mice treated with vehicle or DNase I (red arrows indicate stained vascular wall). Scale bar: 100 μm. (**B**) Representative images of SA-β-gal–stained RMECs treated with PBS, NETs, or NETs pretreated by DNase I for 24 hours. Scale bar: 100 μm. (**C**) The secretion of IL-6, IL-1β, IL-8 and CCL20 from RMECs was detected and measured via ELISA (*n* = 4). (**D**) Flow cytometry analysis of P53, phospho-P53 (p-P53), P16, and P21 in RMECs (*n* = 4). (**E**) The mRNA expression levels of P21, P16, and P53 in retinas from the 2 groups and blank mice were detected via RT-qPCR (*n* = 4). (**F**) Functional protein association network of cGAS (*CGAS*), STING, IRF3, P21, P16, and P53 in humans. (**G**) Flow cytometry analysis of phospho-STING (p-STING) and phospho-IRF3 (p-IRF3) in RMECs 24 hours after treatment (*n* = 4). (**H**) CD69, TNF-α, IFN-γ, and IL-17A expression on human CD4^+^ T cells (CD3^+^CD8^–^) after coculture with RMECs pretreated with PBS, NETs, or NETs plus dasatinib, quercetin, fisetin, or H151 (*n* = 4). Representative data are from at least 3 independent experiments. Data are presented as the mean ± SD. *P < 0.05, **P < 0.01, ***P < 0.001, ****P < 0.0001 for Mann-Whitney test (**E**) and 1-way ANOVA (**C**, **D**, and **G**).

Cell senescence has been verified to cause inflammation and cell dysfunction to participate in various diseases (36). To investigate whether NETs induce retina inflammation through RMEC senescence, we conducted a senescence-associated β-gal (SA-β-gal) assay to visualize senescent cells, which stain blue. Massive blue-stained cells were observed at the retinal vascular wall of EAU mice, while blank mice and DNase I–treated EAU mice showed slight staining of the inner layer (Figure 7A). Besides, NET addition led to an increase in the proportion of SA-β-gal–positive RMECs in vitro, while DNase I downregulated this proportion (Figure 7B). Another obvious feature of senescent cells is that they secrete inflammatory cytokines and chemokines to influence the surrounding microenvironment, which is termed as the senescence-associated secretory phenotype (36). Thus, we tested the supernatant of RMECs using ELISA. We found that DNase I led to a decrease in the augmented inflammatory cytokines IL-6, IL-1β, and IL-8 and chemokine CCL20 secretion induced by NETs (Figure 7C). In addition, flow cytometry analysis and Western blot (WB) assay revealed that NETs induced the expression of P53, phospho-P53, P21, and P16, the key proteins that regulate the senescence process. The upregulation of the aforementioned proteins were all partly reversed by DNase I (Figure 7D and Supplemental Figure 4A). Moreover, these changes were further confirmed by RT-qPCR analysis of the mouse retina. There were increased mRNA levels of P53 (*Trp53*), P21 (*Cdkn1a*), and P16 (*Cdkn2a*) in EAU mouse retina, while DNase I treatment reduced the expression of them (Figure 7E). Above all, these results suggest that NETs may promote RMEC senescence to participate in EAU.

Next, we wondered how NETs induce EC senescence. Recent studies have demonstrated that modulating the cyclic GMP-AMP synthase (cGAS)/stimulator of interferon genes (STING) pathway would influence cell senescence in neurodegeneration and cardiovascular disease in elderly individuals (37, 38). We searched the STRING functional protein interaction database (https://www.string-db.org) and found that cGAS, STING, and its downstream molecule interferon regulatory factor 3 (IRF3) were correlated with the senescence-related molecules P21, P16, and P53 (Figure 7F). To verify this relationship, we stimulated the RMECs and analyzed them via flow cytometry and WB assay. NETs strongly induced the expression of phospho-STING and phospho-IRF3, which indicates the activation of the cGAS/STING pathway (Figure 7G and Supplemental Figure 4B). This tendency was in accordance with NET-induced cell senescence. Altogether, we found that NETs induced RMEC senescence through the cGAS/STING pathway.

Moreover, to verify the role of EC senescence and the cGAS/STING pathway in AU, we treated NETs-stimulated RMECs with 3 classic senolytics (dasatinib, quercetin, and fisetin) and STING antagonist H151. Surprisingly, killing senescent RMECs or inhibiting the STING pathway downregulated CD4^+^ T cell activation and cytokine expression (Figure 7H and Supplemental Figure 5). Overall, we revealed that NETs promoted RMEC senescence via the cGAS/STING pathway to activate Teffs in AU.

## Discussion

In this study, we revealed the regulatory effects of NETs on CD4^+^ T cells in AU and further demonstrated that these effects were mediated by RMECs. On this basis, we showed that RMECs stimulated with NETs exhibited a senescent phenotype. Therefore, we propose for the first time to our knowledge that NETs regulate CD4^+^ T cell function by inducing RMEC senescence to promote AU development. This discovery helps to elucidate the pathogenesis of AU and provides potential therapeutic targets.

AU is believed to be T cell dependent, as it can be arrested by therapies targeting T cells (2). Neutrophils are important effector cells in multiple autoimmune diseases that were also observed to accumulate in eyeballs of patients with uveitis (39). Further investigating the infiltrating cells with animal model EAU, the peak of neutrophils count was found to be associated with the influx of inflammatory CD4^+^ T cells in the retina (6); however, how they function in AU is obscure. When neutrophils are stimulated, they can release NETs to function, which were found to accumulate in the circulation and local lesions of autoimmune diseases (40). Thus, we explored whether neutrophils participate in the pathogenesis of AU through NETs. We found that NETs were higher in patients with active AU than HDs and decreased after effective treatment, which was verified by EAU murine model with neutrophil depletion or DNase I treatment. Moreover, neutrophils from patients with active AU were more prone to produce NETs upon stimulation compared with those from healthy individuals. In contrast, the levels and activity of DNase I showed no significant difference between the uveitis and healthy groups, suggesting that the elevated NETs in AU may be attributed to increased production rather than impaired degradation. This augmented NET production in active uveitis could be due to the inflammatory microenvironment characterized by increased proinflammatory cells, cytokines, complements, and autoantigens, et al., similar to the mechanisms observed in other autoimmune diseases, like SLE, RA, and IBD (40). To conclude, we found that neutrophils promote AU pathogenesis through NETs and that promoting NET degradation could alleviate disease.

NETs, aside from trapping bacteria during infections, can play pathogenic roles in autoimmune diseases through various methods, including promoting Teffs differentiation (13–15). As investigations in EAU have demonstrated that, active CD4^+^ T cells, especially pathogenic Th1 and Th17 cells, were central in EAU induction (2, 4, 31), we investigated whether NETs’ involvement in AU was associated with T cells. We found that deletion of neutrophils or degeneration of NETs with DNase I could reduce the infiltration of activated CD4^+^ T cells, Th1 cells, and Th17 cells both locally and systematically in EAU, thereby alleviating disease severity. As deletion of neutrophils can lead to serious infections, the use of DNase I allows for more specific and safer treatment of AU. Our findings reveal that the progression of AU is facilitated by NETs via the regulation of CD4^+^ T cells and that targeting NETs could make a potential therapeutic strategy for treating AU.

BRB breakdown leads to an increase in vascular permeability and a consequent influx of inflammatory cells to promote the development of AU (3, 5). Yang et al. suggested that neutrophils infiltrate around retinal blood vessels and neutrophil inhibition could regulate retinal vascular permeability in EAU (39). In addition, NETs were reported to open the vascular barrier to allow melanoma cell dissemination and blood cell leakage by regulating adhesion molecules and intracellular junctional proteins on ECs (41, 42). These studies indicate the intricate relationship between NETs and the BRB in AU. Based on this research, we hypothesize that NETs may destroy BRB integrity to allow inflammatory cell influx, resulting in AU development. In our studies, we found that an increase in NETs was accompanied by BRB breakdown in EAU mice and in vitro BRB model. This could be owing to our findings in this study that NETs stimulated adhesion molecule expression on RMECs, as RMECs are the main component of BRB. Thus, these findings clarify that NETs promote AU by destroying the integrity of retinal blood vessels.

Beyond maintaining structure of blood vessels, ECs also exert immunomodulatory effects. Adhesion molecules, which are strongly expressed on the BRB during uveitis, are known to mediate leukocyte adhesion in addition to maintaining BRB integrity (35, 43). Here, we showed the upregulation of adhesion molecules and selectin in EAU retina and further revealed that RMECs stimulated by NETs were more prone to adhere to CD4^+^ T cells, indicating that NETs could facilitate CD4^+^ T cell adherence to RMECs to participate in AU. In addition, ECs could act as semiprofessional antigen-presenting cells. Once CD4^+^ T cells are attached to ECs, ECs that express MHC-II and costimulatory molecules are able to activate T cells (19). Previous studies have revealed the upregulation of antigen presentation–related genes in EAU retinal ECs (44, 45), which was confirmed by our study. Moreover, we demonstrated NETs’ ability to stimulate MHC-II and costimulatory molecules in RMECs and DNase I’s therapeutic effect both in vivo and in vitro. Building upon the discovery, we extended our investigation with an in vitro coculture system to demonstrate that the RMECs stimulated by NETs are capable of CD4^+^ T cell activation and inflammatory cytokine secretion. Thus, it may be concluded that NETs activate ECs to promote AU development.

Interestingly, we found that the RVECs activated by NETs become senescent. Senescent cells are highly proinflammatory cells induced by pathological stimuli (46). There is a close relationship between cell senescence and the ECs phenotype changes that we indicated above, supported by previous studies showing that senescent ECs express increased adhesion molecules, secrete inflammatory cytokines, and impair barrier integrity to participate in chronic inflammatory diseases (36, 47). Meanwhile, the cGAS/STING pathway, which participates in immune responses through sensing pathogenic DNA (48, 49), can lead to cell senescence (37, 38). In this study, we found surprisingly that NETs could activate the cGAS/STING pathway in RMECs in company with senescent cell accumulation. This phenomenon could be partly abrogated by removing the DNA component with DNase I, which may be attributed to DNA being the essential component of NETs. In addition, treatment with senolytic drugs or a STING antagonist alleviated Teff activation and cytokine expression induced by NET-treated RMECs. Therefore, EC senescence could be a mechanism through which NETs regulate CD4^+^ T cells to mediate disease progression.

In summary, our work highlights the pathogenic role of NETs in AU through promoting RMEC senescence to activate CD4^+^ T cells and represents a link between innate and adaptive immunity to further enrich our understanding of AU pathogenesis. Therefore, targeting NETs may be therapeutic for AU.

## Methods

### Sex as a biological variable.

In our human studies, both male and female participants were included, with no clear sex-related differences observed. In the mouse studies, only female mice were used. Previous research has indicated no significant difference in susceptibility to EAU between male and female mice (50). Therefore, sex was not considered a biological variable, and the findings are expected to be relevant for more than one sex.

### Patient enrollment.

This study recruited 20 patients over 18 years of age with active noninfectious panuveitis from Zhongshan Ophthalmic Center of Sun Yat-sen University. The inclusion criteria required active disease characterized by presence of anterior chamber cell, vitreous cell (graded referring to the Standardization of Uveitis Nomenclature [SUN] criteria, ref. 51), and retinal vasculitis observed via fundus fluorescein angiography in at least 1 eye. All patients were diagnosed with idiopathic uveitis with only eyes involved or Behcet’s uveitis following the guidelines of the Behcet’s Syndrome Research Committee of Japan (52–54). Patients with any other known infectious disease or systemic inflammatory disease, except for Behcet’s disease, were excluded. Additionally, none of the patients had received systemic immunosuppressive medications or prednisone for at least 1 month before blood sampling. Detailed patient information is provided in Supplemental Table 1. The longitudinal comparison between the active and inactive/remission phases (as defined by SUN criteria) was conducted with detailed information for both phases provided in Supplemental Table 2.

### Clinical sample collection.

Blood was drawn from patients with uveitis and age- and sex- matched HDs. Briefly, 10 mL venous blood was obtained and centrifuged to isolate the plasma. Then, the remaining blood was mixed with 0.9% sodium chloride solution and layered onto Ficoll (Cytiva, 17144003) for density gradient centrifugation according to the manufacturer’s instructions. CD4^+^ T cells were enriched from the peripheral blood mononuclear cell layer by magnetic bead separation (Stemcell, 17952). The mixture of neutrophils and red blood cells precipitates was in the lowest layer. Briefly, 10 mL red blood cell lysis buffer (Biosharp, BL503A) was mixed with the cell pellet to allow for cell lysis for 5 minutes and centrifuged at 500*g* for 5 minutes. After removing the supernatants, pellets enriched with neutrophils were ready to use. The cell liveness was >99%, and the purity was >94%, as assessed by flow cytometry (BD LSRFortessa) (Supplemental Figure 6).

### NET production and isolation.

Isolated neutrophils were resuspended in RPMI 1640 medium, and 2 × 10^6^ cells/well were seeded in 6-well plates. To collect NETs, cells were stimulated with 25 nM PMA (Sigma, P8139) for 4 hours, the supernatant was discarded, and the wells were rinsed carefully with ice-cold PBS. Then, PBS was added, collected, and centrifuged at 400*g* for 10 minutes to discard the neutrophils and cell debris, and NETs were left in the supernatants (12, 24). Isolated NETs were used immediately or stored at –80°C until use.

### Quantification of cfDNA and MPO-DNA complexes.

cfDNA in human plasma, mouse serum, neutrophil supernatants, and isolated NETs was detected with a Quant-iT Picogreen dsDNA Kit (Invitrogen, P11496). Pure cfDNA standards and samples were incubated with reagent from the kit, and the amount of DNA was measured according to the fluorescence signals detected with a microplate fluorescence reader (Synergy H1mfd, BIOTEK). MPO-DNA complexes were identified as described previously (21, 55). A 96-well plate was coated with 5 μg/mL anti-MPO monoclonal antibody (R&D Systems, AF3667) overnight at 4°C. After blocking in incubation buffer, samples were added with the peroxidase-labeled anti-DNA monoclonal antibody from the Cell Death Detection ELISA kit (Roche, 11774425001) and incubated at room temperature for 2 hours. After 3 washes with PBS, peroxidase substrates were added and incubated for 20 minutes at 37°C in the dark. The absorbance was measured at a wavelength of 450 nm by a microplate reader (Synergy H1mfd, BIOTEK).

### DNase I level and activity.

DNase I level of human plasma and mouse serum was detected through the sandwich ELISA assay method (LifeSpan BioSciences, LS-F16113 and LS-F4463) according to the manufacturers’ instructions. DNase I activity was detected with a DNase I Activity Fluorometric Assay Kit (Beyotime, P0345M).

### Visualization of NETs.

Isolated neutrophils (5 × 10^5^ cells/wells) were seeded onto poly-L-lysine–coated coverslips in 24-well plates and stimulated by PBS, 5 nM recombinant human complement C5a (MedChemExpress, HY-P700862), or 25 μM PMA for 4 hours. The cells were washed and fixed with 4% paraformaldehyde for 15 minutes. Then, they were permeabilized with 0.5% Triton X-100 for 10 minutes and blocked with 3% BSA for 1 hour for further staining. Afterward, the coverslips were incubated with 10 μg/mL goat anti-MPO (R&D Systems, AF3667) overnight. Paraffin-embedded mouse eyeball sections were deparaffinized, dipped in antigen retrieval solution, permeabilized with 0.5% Triton X-100, and blocked with 3% BSA. Then, they were incubated with 10 μg/mL goat anti-MPO (R&D Systems, AF3667), rabbit anti-H3Cit (1:50, Abcam, ab5103), or rat anti-NE (1:100, R&D Systems, MAB4517) at 4°C overnight. Alexa Fluor–conjugated secondary antibodies (1:500, Invitrogen, A-21432, A32731, A-21434) were incubated the next day for 2 hours, followed by DAPI (Abcam, ab104139) to stain the nuclei. Images were obtained by laser scanning confocal microscopy (LSM880, Zeiss).

### Mice.

Female C56BL/6j mice (6–8 weeks, 20–25 g) were supplied by the GuangDong Yaokang Biotechnology Company and maintained in a specific pathogen–free environment.

### Induction and treatment of EAU.

Mice were injected subcutaneously with 100 μL hIRBP_1–20_ (GPTHLFQPSLVLDMAKVLLD, 2 μg/mL; Sangon Biotech) emulsified in 100 μL incomplete freund’s adjuvant (Sigma, F5506), which contained the mycobacterium tuberculosis strain H37Ra (2.5 mg/mL; BD Difco). Additionally, 200 ng pertussis toxin (List Labs, #180) was administered intraperitoneally twice (on day 0 and day 2) to each mouse after immunization. Neutrophils were depleted by intraperitoneally injecting 10 μg/g of the neutrophil-specific antibody Ly6G (Biolegend, 127649) or 10 μg/g rat IgG2A isotype control antibody (Biolegend, 400565) as isotype control for every 2 days (39). NET degradation was performed by daily subcutaneous administration of 150 U/mouse DNase I (Roche, 04536282001) in 0.9% sodium chloride solution or 0.9% sodium chloride solution as vehicle every day.

### EAU evaluation.

Mouse fundus photographs and fluorescein angiograms were taken with a Micron IV Retinal Imaging Microscope (PHOENIX) on day 14 after immunization. Mice were injected intraperitoneally with 100 μL 4% fluorescein sodium (Sigma, 46955) (56). Clinical scores were graded based on criteria reported previously (57). After that, the eyes were fixed, dehydrated, embedded, sectioned, and stained with H&E or SA-β-gal. The H&E-stained sections were photographed with a positive fluorescence microscope (Nikon ECLIPSE Ni-U) and graded in compliance with previously described criteria (57).

### Isolation of mouse serum and retinal cells.

Mouse blood from the caudal vein was collected, stewed, and centrifuged for serum collection and the NET component detection. The eyeballs were washed twice with PBS after systemic perfusion. The retinas were dissected from eyeballs under a microscope, ground carefully, and filtered through cell strainers for single-cell suspension. The cells were then resuspended and stained for flow cytometry analysis.

### RMEC treatment.

Primary RMECs were obtained from Shanghai Zhong Qiao Xin Zhou Biotechnology Company (PRI-H-00142) and cultured in ECM medium (ScienCell, 1001) supplemented with 1% growth factor, 1% penicillin, and 5% FBS. When the cells were grown to 80%–90% confluence, they were incubated with PBS, 0.15 μg/mL NETs, 0.15 μg/mL NETs pretreated with 100 U/mL DNase I (37°C, 1 hour) (41, 42, 58), dasatinib (100 nm, MedChemExpress, HY-10181) (59), quercetin (10 μm, MedChemExpress, HY-18085) (60), fisetin (10 μm, MedChemExpress, HY-N0182) (61), or H151 (10 μm, MedChemExpress, HY-112693) (62) for different durations according to experimental requirements.

### T cell adhesion and activation assays.

RMECs were cultured in 24-well plates and treated for 24 hours as described above. Isolated human CD4^+^ T cells (2 × 10^5^ cells/well) were cocultured with RMECs in RPMI 1640 medium supplemented with 10% FBS and dynabeads human T-activator CD3/CD28 (Gibco, 11161). For the adhesion assay (63, 64), supernatants were discarded, and the platelets were rinsed with PBS after coculture for 24 hours. After 4% paraformaldehyde fixation and DAPI staining, T cells adhered to RMECs were imaged with a Nikon inverted microscope (Nikon Corporation Eclipse Ts2FL) and counted with ImageJ (NIH). Moreover, the coculture lasted 6 hours for flow cytometry analysis of CD69 or 24 hours for intracellular cytokine expression.

### In vitro permeability assay.

The RMECs were grown to confluence in a Transwell upper chamber with 0.4 μm pore size membrane (Corning Life Sciences). The cells were treated as described above for 24 hours and washed with PBS. The bottom chamber was filled with medium alone. FITC-conjugated dextran (1 mg/mL, Sigma, FD40S) was added to the upper chamber, and the fluorescence intensity of FITC-dextran that passed through the endothelial layer into the bottom chamber was measured after 1 hour with a microplate fluorescence reader (Synergy H1mfd, BIOTEK).

### SA-β-gal.

For determination of SA-β-gal activity, mouse eyeball sections and RMECs subjected to different treatments were washed twice in PBS and stained using a Senescence β-Galactosidase Staining Kit (Beyotime, C0602) in accordance with the manufacturers’ instructions. Images were taken with a positive fluorescence microscope.

### Flow cytometry.

Single cells were isolated from mouse retinas, mouse spleens, human PBMCs, or RMECs. They were stained with the antibodies (obtained from Biolegend if not specified) listed below: Zombie NIR (423105), anti-mouse CD45 (103248), anti-mouse CD4 (100434), anti-mouse CD69 (104508), anti-mouse CD25 (102016), anti-mouse CD31 (eBioscience, 11-0311-81), anti-mouse CD80 (104733), anti-mouse CD86 (105012), and anti-mouse MHC-II (107639), anti-human CD45 (304048), anti-human CD16 (980112), anti-human CD66b (305117), anti-human CD3 (317310), anti-human CD8 (300934), anti-human CD69 (310934), anti-human CD31 (303132), anti-human CD80 (305207), anti-human CD86 (305412), anti-human HLA-DR (327018), anti-human ICAM-1 (353105), anti-human VCAM-1 (305815), and anti-human E-selectin (384103). Due to trypsin sensitivity, the staining of E-selectin and VCAM-1 on RMECs was conducted before the digestion of 0.2% Trypsin-EDTA (Gibco) according to Gräbner et al. (65). The other proteins on RMECs were labeled after trypsinization (66, 67). The gating strategy of human and mouse cells was shown in Supplemental Figures 6 and 7.

For T cell intracellular cytokine staining, Cell Stimulation Cocktail (eBioscience, 00-4975-93) was added for 5 hours of activation. After cell surface marker staining for 20 minutes, fixation for 1 hour, and permeabilization for 30 minutes, the intracellular cytokine staining (Biolegend) with anti-mouse IFN-γ (505836), anti-mouse IL-17A (506926), anti-mouse Foxp3 (eBioscience, 11-5773-82), anti-human IFN-γ (506507), anti-human IL-17A (512310), and anti-human TNF-α (376209) was conducted. For RMEC intracellular cytokine staining, after surface fluorescent labeling, fixation, and permeabilization, the cells were incubated with anti-human P53 (Invitrogen, MA1-19551), anti-human phospho-P53 (Invitrogen, MA5-36928), or anti-human PSTING (Cell Signaling Technology, 43499) antibodies for direct labeling. For indirect labeling, the cells were first incubated with anti-human P16 (Cell Signaling Technology, 18769), anti-human P21 (Cell Signaling Technology, 2947), or anti-human pIRF3 (Cell Signaling Technology, 29047) antibodies overnight in 4°C and then with Alexa Fluor–conjugated secondary antibodies (Invitrogen, F-2765 and P-2771) overnight at 4°C the next day. The stained cells were collected on a BD LSRFortessa instrument (BD Biosciences) and analyzed with FlowJo software 10.8.1 (FlowJo Company).

### ELISA.

Mouse retina was separated, collected on ice, weighed, and added to PBS (9 mL PBS per 1 g tissue), and then an appropriate amount of protease inhibitor was added. Then, the mixture was ground with a high-speed low-temperature tissue grinding machine (Servicebi), centrifuged at 5,000*g* for 5 minutes, and the supernatant was separated and frozen at –80°C. The concentrations of mouse TNF-α (Elabscience, E-HSEL-M0009), IL-1β (Ebioscience, E-HSEL-M0001), and CCL2 (Thermo Fisher, 88-7391-22) were measured with a microplate reader. RMECs were treated for 24 hours as described above in 12-well plates, rinsed twice with PBS, and then cultured in 5% ECM. After 24 hours, the supernatants were collected, centrifuged to remove cell debris, and stored at –80°C. The concentrations of human IL-6 (Invitrogen, 88-7066-88), IL-8 (Invitrogen, 88-8086-88), IL-1β (Invitrogen, 88-7261-88), and CCL20 (RayBiotech, P78556) were measured with microplate reader (Bio-Tek).

### RT-qPCR.

Total RNA from mouse retinas and RMECs was extracted using the RNA-Quick Purification Kit (EScience, RN001) and measured with a NanoDrop spectrophotometer (ND-100, NanoDrop Technologies). Then, the RNA was reverse-transcribed into cDNA with PrimeScript RT Master Mix (TaKaRa). RT-qPCR was conducted with SYBR Premix ExTaq II (TaKaRa). The relative mRNA expression levels of TNF-α, IL-1β, and CCL2 (Sangon Biotech) were analyzed with the 2^–ΔΔCt^ method.

### WB assay.

Protein lysates were prepared from cell samples using lysis buffer containing protease and phosphatase inhibitors (KeyGEN BioTECH, KGB5303). Proteins were separated by SDS-PAGE and transferred onto PVDF membranes. The membranes were blocked with 5% BSA in TBS-Tween for 2 hours at room temperature, followed by incubation with primary antibodies obtained from Cell Signaling Technology overnight at 4°C: STING (13647), phospho-STING (19781), IRF3 (11904), Phospho-IRF3 (29047), Phospho-p53 (9284), P21 (2947), P16 (80772), P53 (2524), or β-actin (4970). After washing, membranes were incubated with HRP-conjugated secondary antibodies (Cell Signaling Technology, 7074 and 7076) for 2 hours at room temperature. Detection was performed using enhanced chemiluminescence reagents, and the signals were captured with an imaging system and analyzed by ImageJ (NIH).

### Statistics.

For comparisons between 2 groups, 2-sided Student’s unpaired 2-tailed *t* test was used when the sample size was greater than 15 and the data were normally distributed; otherwise, the Mann-Whitney test was employed. For longitudinal comparisons of NETs in active- and stable-phase patients, nonparametric paired 2-tailed *t* test was used. One-way ANOVA was utilized for comparisons among 3 groups followed by pairwise comparison, corrected for multiple comparisons. Further statistical details of experiments can be found in figure legends. Data are presented as mean ± SD. *P* < 0.05 was considered to indicate a statistically significant difference. All the statistical analyses were performed using GraphPad Prism version 10.

### Study approval.

Informed consents forms were obtained in accordance with the Declaration of Helsinki and approved by the Institutional Review Board of Zhongshan Ophthalmic Center in Guangzhou China (2020KYPJ104). This study was approved by the Ethics Committee of Zhongshan Ophthalmic Center of Sun Yat-sen University, and written informed consent was obtained from all participants. Animal studies were approved by the Institutional Animal Care and Use Committee of Zhongshan Ophthalmic Center Sun Yat-sen University in Guangzhou (O2021025). All the experiments were performed in accordance with the Association for Research in Vision and Ophthalmology (ARVO) Statement for the Use of Animals in Ophthalmic and Vision Research.

### Data availability.

Values for all data points in graphs are reported in the Supporting Data Values file. All additional data are provided in the supplement and are available from the corresponding author.

## Author contributions

Zuoyi Li, Zhuang Li, and YH designed the study, analyzed the data, and wrote the manuscript. YX and YS helped to revise the manuscript. GC, ZX, JH, and WZ assisted with the experimental methodology. MW, JC, and HH provided guidance on the individual experiments and supervised the study. XC and DL were responsible for the design and revision of this manuscript and the approval of the final manuscript. The order of co–first authors was determined based on contributions to the experiments and draft writing. All the authors read and approved the final manuscript.

## Supplementary Material

Supplemental data

Unedited blot and gel images

Supporting data values

## Version 1 01/23/2025

Electronic publication
