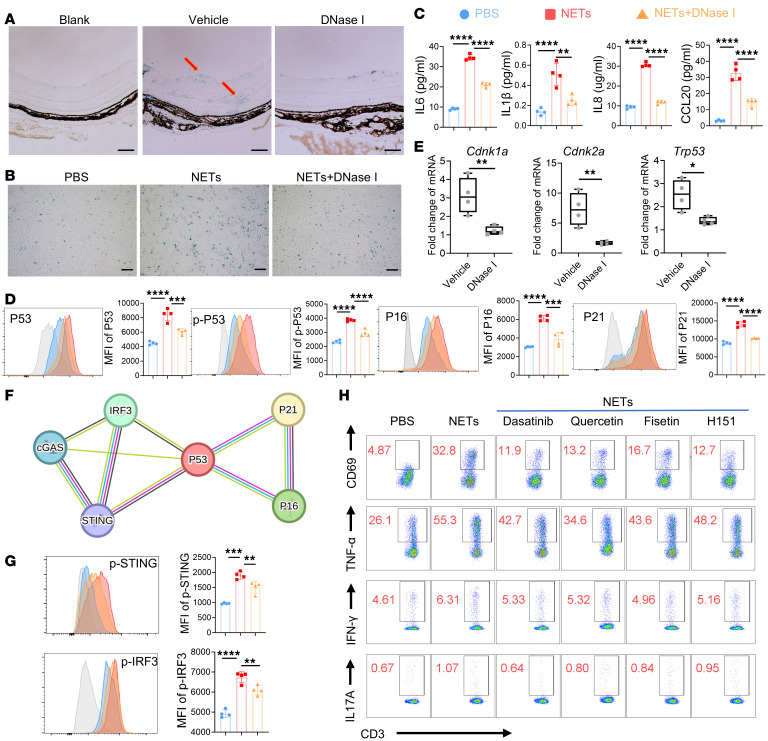# Corrigendum to Neutrophil extracellular traps potentiate effector T cells via endothelial senescence in uveitis

**DOI:** 10.1172/jci.insight.202699

**Published:** 2026-01-09

**Authors:** Zuoyi Li, Zhuang Li, Yunwei Hu, Yanyan Xie, Yuxun Shi, Guanyu Chen, Jun Huang, Zhiqiang Xiao, Wenjie Zhu, Haixiang Huang, Minzhen Wang, Jianping Chen, Xiaoqing Chen, Dan Liang

Original citation: *JCI Insight*. 2025;10(2):e180248. https://doi.org/10.1172/jci.insight.180248

Citation for this corrigendum: *JCI Insight*. 2026;11(1):e202699. https://doi.org/10.1172/jci.insight.202699

After publication, the authors became aware that the CD3^+^CD69^+^ flow cytometry plots for dasatinib and quercetin in Figure 7H were inadvertently transposed during figure assembly. The correct figure panels are shown below. The HTML and PDF versions have been updated online.

The authors regret the error.

## Figures and Tables

**Figure 7:**