# Supplementary material for: Neutrophil extracellular traps potentiate effector T cells via endothelial senescence in uveitis
Source: JCI Insight. 2025 Jan 23;10(2):e180248. doi: 10.1172/jci.insight.180248 (PMC11790022; doi:10.1172/jci.insight.180248)
Supplement: Supplemental data [file jciinsight-10-180248-s213.pdf]

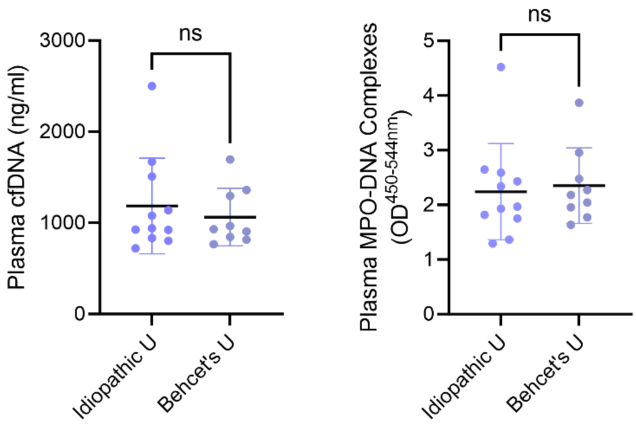

Supplemental Figure 1 NETs between idiopathic and Behcet’s uveitis patients.

CfDNA and the MPO-DNA complex were detected in the plasma of idiopathic uveitis (n=11) and Behcet's uveitis patients (n=9). Data are presented as the mean±SD. $^{ns}p>0.05$ for Mann-Whitney test.

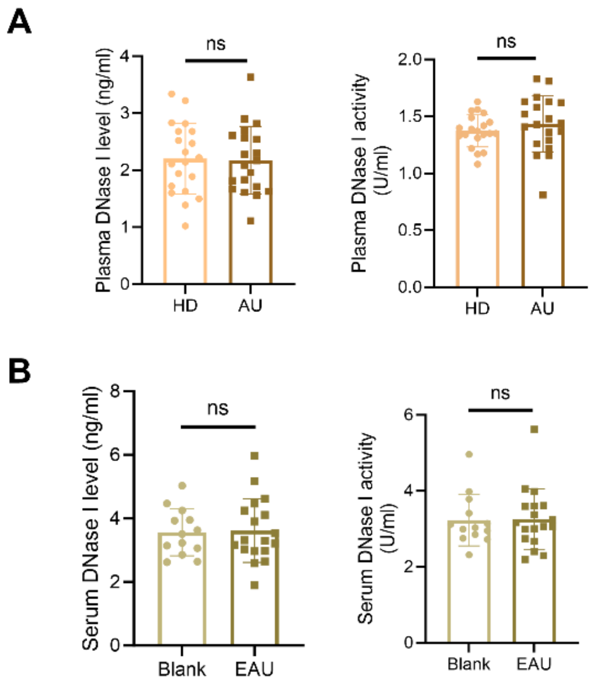

Supplemental Figure 2 Detection of DNase I level and activity from human plasma and mouse serum.

(**A**) DNase I level and activity were detected in the plasma of AU patients (n=20) and HD (n=20). (**B**) Serum from EAU (n=18) and blank (n=13) mice was also collected for DNase I level and activity detection. Data are presented as the mean±SD. $^{ns}p>0.05$ for student's unpaired t test (**A**) and Mann-Whitney test (**B**).

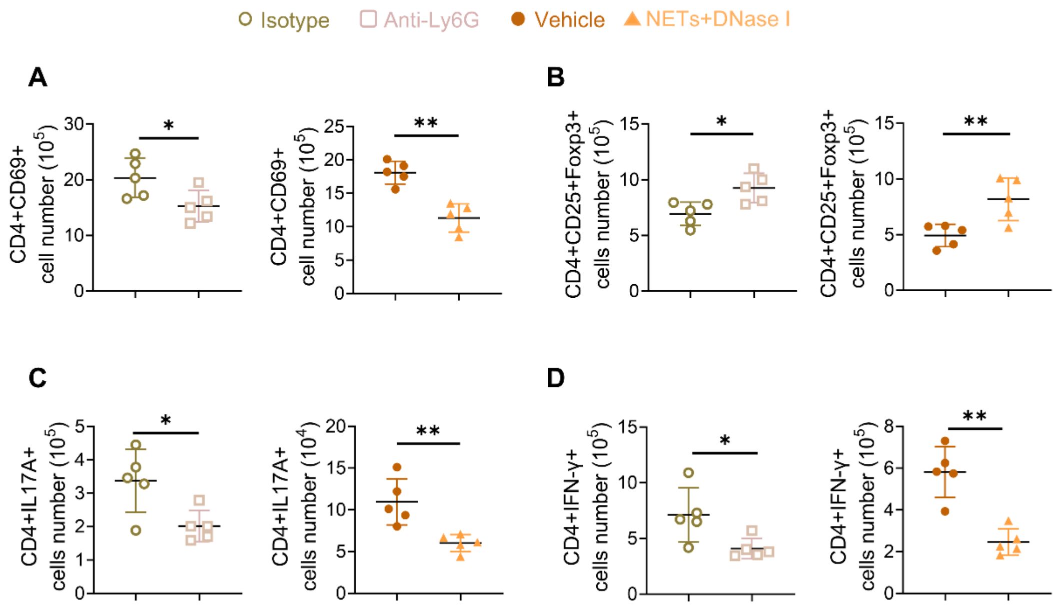

Supplemental Figure 3 Anti-Ly6G antibody and DNase I regulated systemic immune cells.

(**A-D**) Lymphocytes from the spleen were calculated to evaluate the number of early activated CD4+T cells (CD4+CD69+), Tregs (CD4+CD25+Foxp3+), Th17 cells (CD4+IL17A+) and Th1 cells (CD4+IFN-γ+) from EAU mice treated with anti-Ly6G antibody or DNase I with their control on day 14 after immunization (n=5). Representative data from at least three independent experiments. Data are presented as the mean±SD. *$p<0.05$, **$p<0.01$ for Mann-Whitney test (**A-D**).

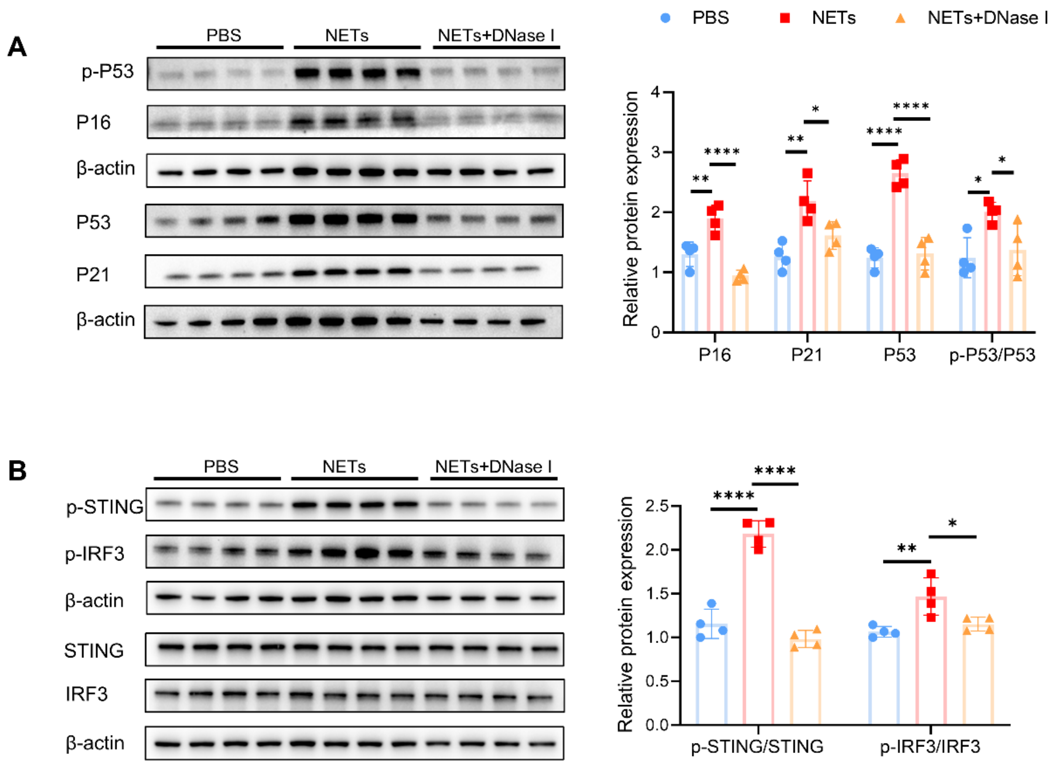

Supplemental Figure 4 NETs regulated the senescence process of RMECs.

(**A**) The level of p-P53, P16, P53, P21 and β-actin in RMECs were detected by WB assay and their statistical graphs were shown (n=4). (**B**) The level of p-STING, p-IRF3, STING, IRF3 and β-actin in RMECs were detected by WB assay and their statistical graphs were shown (n=4). Representative data from at least three independent experiments. Data are presented as the mean±SD. *$p<0.05$, **$p<0.01$, ***$p<0.001$, ****$p<0.0001$ for one-way ANOVA (**A** and **B**).

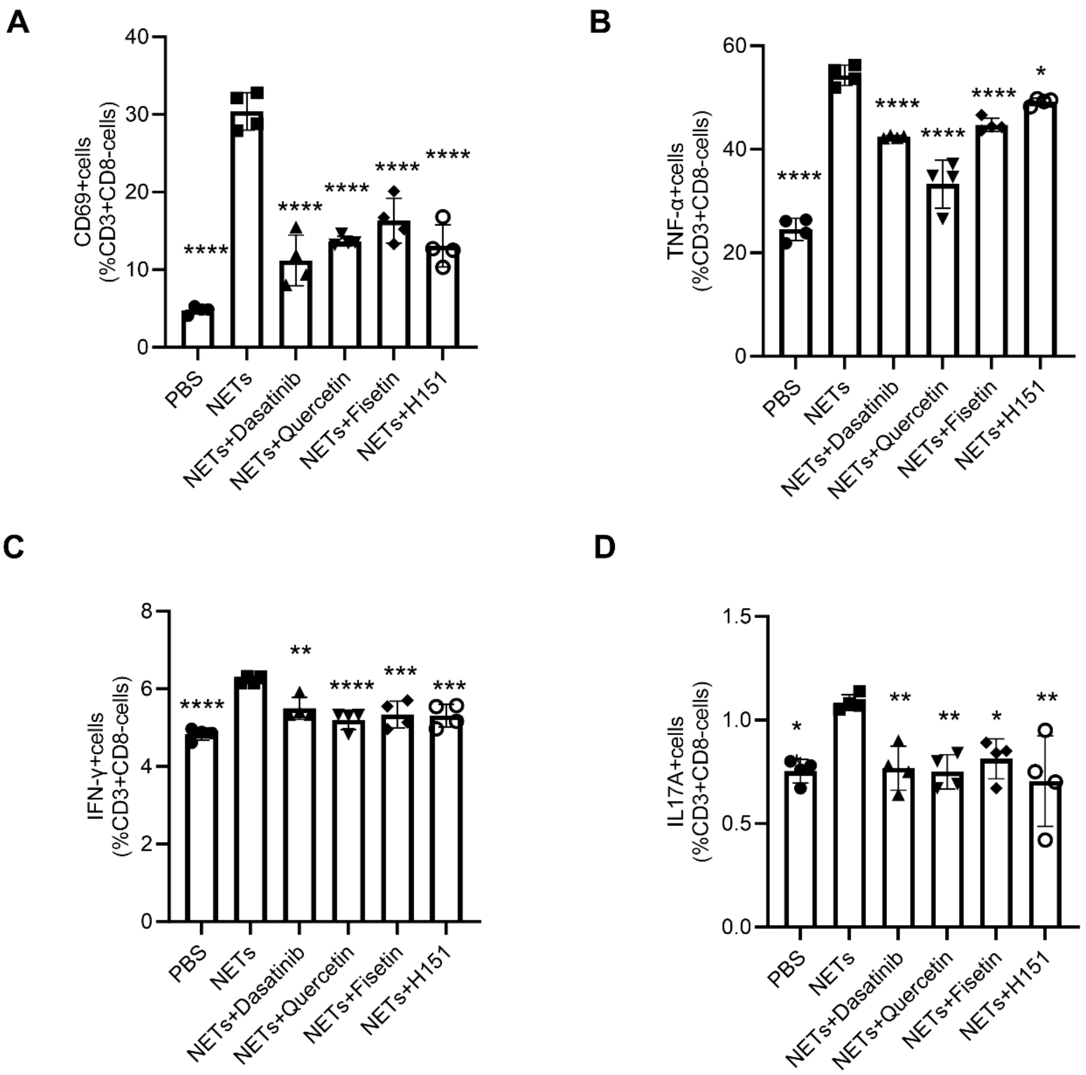

Supplemental Figure 5 The statistic graphs of immune cell frequencies after medication.

(**A-D**) The statistic graphs of CD69, TNF-α, IFN-γ and IL17A expression on human CD4+ T cells (CD3+CD8-) after coculture with RMECs pretreated with PBS, NETs, or NETs plus dasatinib, quercetin, fisetin or H151 (n=4). Representative data from at least three independent experiments. Data are presented as the mean±SD. *p<0.05, **p<0.01, ***p<0.001, ****p<0.0001 for one-way ANOVA (**A-D**).

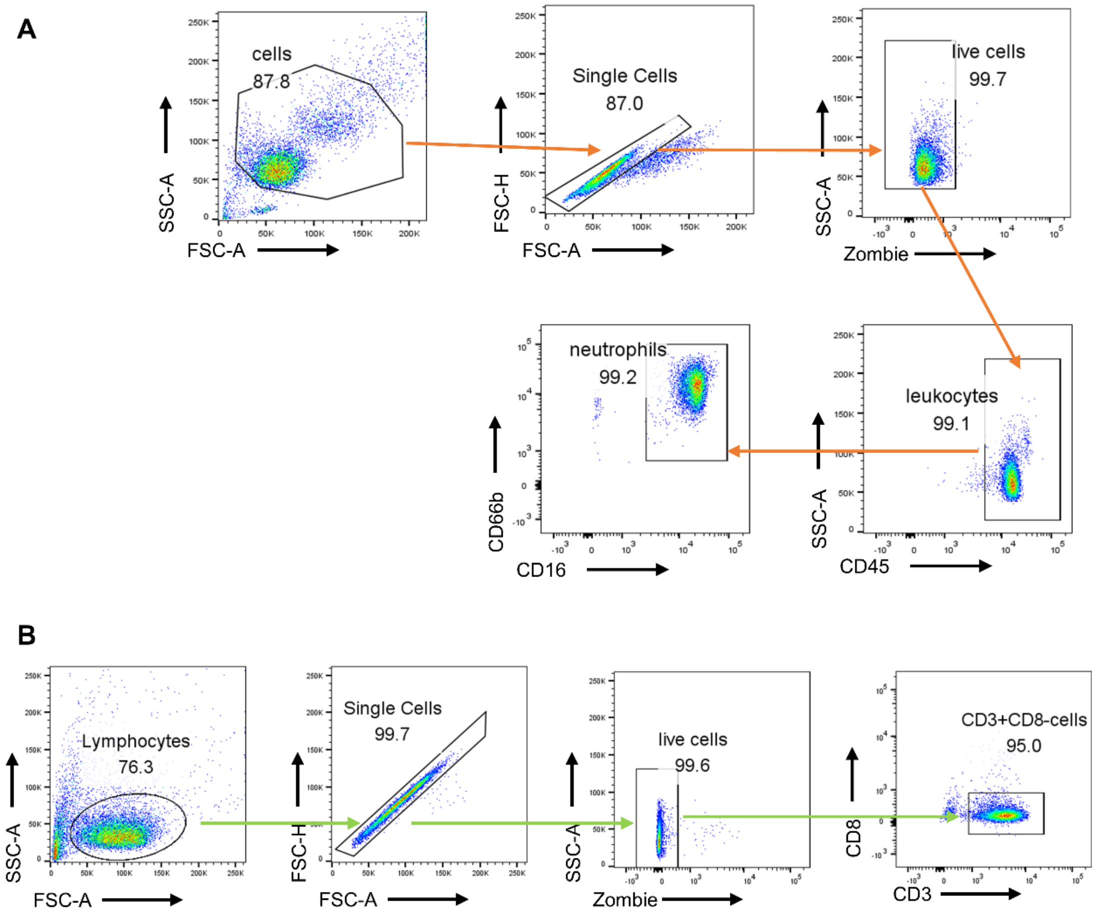

Supplemental Figure 6 Human cell flow cytometry gating strategy.

(**A**) Gating strategy of neutrophils from human blood. (**B**) Gating strategy of sorted human CD4+ T cells.

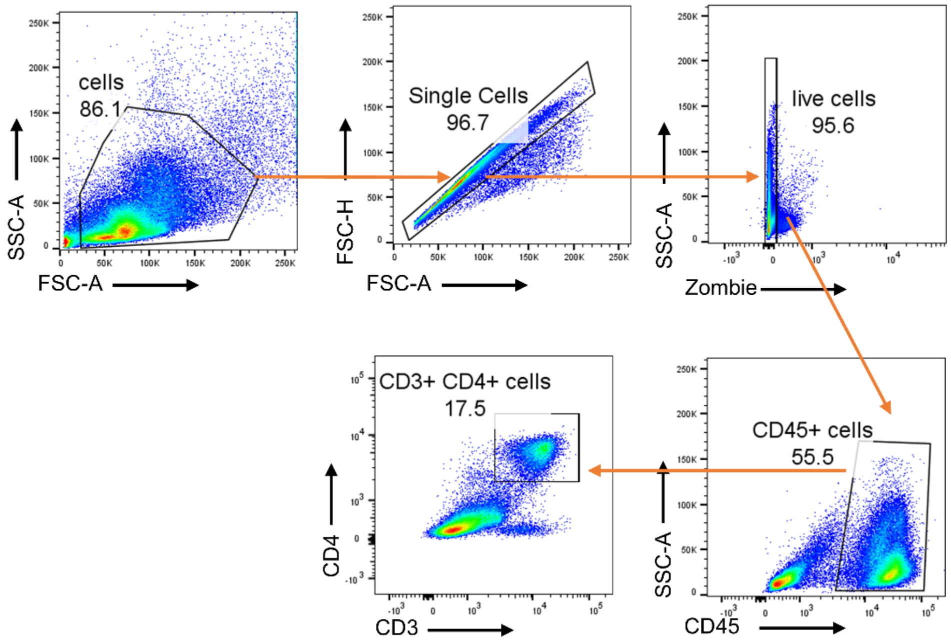

Supplemental Figure 7 Mouse cell flow cytometry gating strategy.

Gating strategy of CD4+ T cells from mouse spleen.

Supplemental Table 1 Clinical characteristics of active AU patients.

No.	Age (yrs)	Sex	Context	Keratic precipitate[A]	Anterior chamber cell[A]	Vitreous cell[A]	Diagnosis
1	33	M	Newly diagnosed, untreated	-	+	++	Idiopathic U
2	27	M	Relapse, off therapy	++	++	+++	Behcet's U
3	41	M	Relapse, off therapy	+	+	++	Idiopathic U
4	38	F	Relapse, off therapy	-	++	+	Idiopathic U
5	23	M	Relapse, off therapy	+	Hypopyon	+++	Behcet's U
6	24	M	Relapse, off therapy	+	++	+	Idiopathic U
7	18	M	Relapse, off therapy	++	+	+	Behcet's U
8	42	M	Newly diagnosed, untreated	+	+++	+++	Idiopathic U
9	40	F	Relapse, off therapy	-	++	++	Behcet's U
10	19	F	Newly diagnosed, untreated	++	+++	++	Idiopathic U
11	39	F	Newly diagnosed, untreated	+	Hypopyon	+++	Behcet's U
12	35	F	Newly diagnosed, untreated	++	+++	++++	Idiopathic U
13	43	F	Relapse, off therapy	+	++	+	Behcet's U
14	29	F	Relapse, off therapy	++	++	++++	Behcet's U
15	20	F	Newly diagnosed, untreated	+	+	++	Idiopathic U
16	22	F	Relapse, off therapy	+	Hypopyon	+++	Behcet's U
17	32	M	Newly diagnosed, untreated	++	++	++	Idiopathic U
18	51	M	Newly diagnosed, untreated	+	+	+	Behcet's U
19	44	F	Newly diagnosed, untreated	+	+	+	Idiopathic U
20	35	M	Relapse, off therapy	++	+++	+	Idiopathic U

F, female; M, male; U, uveitis. [A]The keratic precipitate, anterior chamber cell and vitreous cell of the heavier eye was recorded, '-' indicates none, and '+' denotes increasing numbers.

Supplemental Table 2 Clinical characteristics of AU patients in active and remission/inactive phase.

No.	Sex	Age (yr)	Diagnosis	Context and medication	Keratic precip-itate[A]	Anterior chamber cell[A]	Vitreous cell[A]
1	M	42	Idiopathic U	Relapse, off therapy	-	-	++
				Inactive： MMF 0.75mg bid	-	-	-
2	M	34	Behcet's U	Relapse, off therapy	++	++	++
				Inactive： ADA 40mg q2w	-	-	-
3	F	25	Idiopathic U	Remission	-	-	-
				Relapse, off therapy	-	++	++
4	F	43	Behcet's U	Relapse, off therapy	-	+	++
				Inactive： MMF 0.75g bid; ADA 40mg q2w; prednisone 10mg qd	-	-	-
5	F	36	Idiopathic U	Newly diagnosed, untreated	+	+	+
				Remission	-	-	-
6	F	20	Idiopathic U	Relapse, off therapy	+	+	++
				Inactive: MTX 15mg qw; ADA 40mg q2w	-	-	-
7	M	36	Behcet's U	Relapse, off therapy	++	Hypopyon	++
				Inactive: MMF 0.75g bid; prednisone 10mg qd	-	-	-
8	F	18	Idiopathic U	Relapse, off therapy	+	+++	+
				Inactive: MTX 15mg qw; ADA 40mg q2w	-	-	-
9	M	50	Idiopathic U	Relapse, off therapy	-	+	++
				Inactive: CsA 0.75g bid; prednisone 2.5mg qd; ADA 40mg q2w	-	-	-
10	F	25	Behcet's U	Newly diagnosed, untreated	++	+	++
				Inactive: MTX 15mg qw; ADA 40mg q2w	-	-	-
11	M	19	Idiopathic U	Newly diagnosed, untreated	+	++	++
				Inactive: MTX 15mg qw; ADA 40mg q2w	-	-	-
12	M	53	Idiopathic U	Newly diagnosed, untreated	++	+	++
				Inactive: CsA 0.5g bid; prednisone 10mg qd; ADA 40mg q2w	-	-	-
13	M	38	Behcet's U	Relapse, off therapy	+	++	++
				Inacitve: ADA 40mg q2w; prednisone 10mg qd; AZA 50mg tid	-	-	-

ADA, adalimumab; AZA, Azathioprine; bid, twice per day; CsA, Cyclosporin A; F, female; M, male; MMF, mycophenolate mofelil; MTX, methotrexate; qd, once per day; qw, once per week; q2w, once per two weeks; tid, three times per day; U, uveitis. [A]The keratic precipitate, anterior chamber cell and vitreous cell of the heavier eye was recorded, '-' indicates none, and '+' denotes increasing numbers.
