# Supplementary material for: Neutrophil extracellular traps potentiate effector T cells via endothelial senescence in uveitis
Source: JCI Insight. 2025 Jan 23;10(2):e180248. doi: 10.1172/jci.insight.180248 (PMC11790022; doi:10.1172/jci.insight.180248)
Supplement: Unedited blot and gel images [file jciinsight-10-180248-s214.pdf]

Full unedited blot for Supplemental Figure 4A

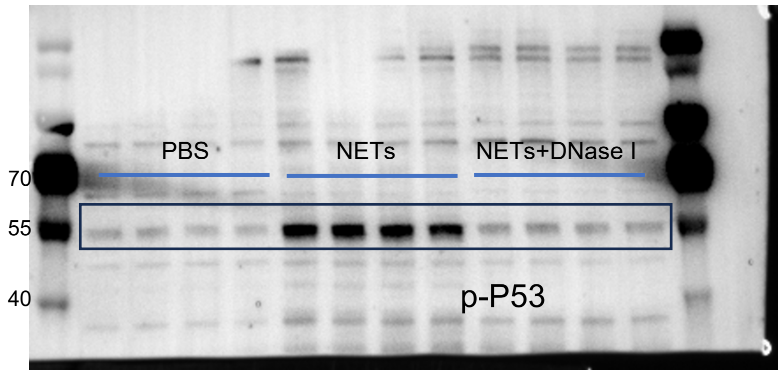

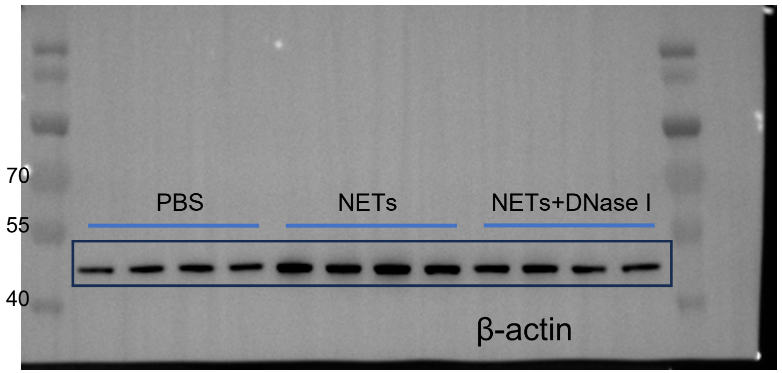

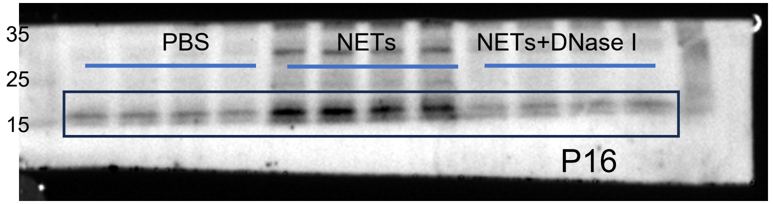

These lanes were noncontiguous on the same gel.

Full unedited blot for Supplemental Figure 4A

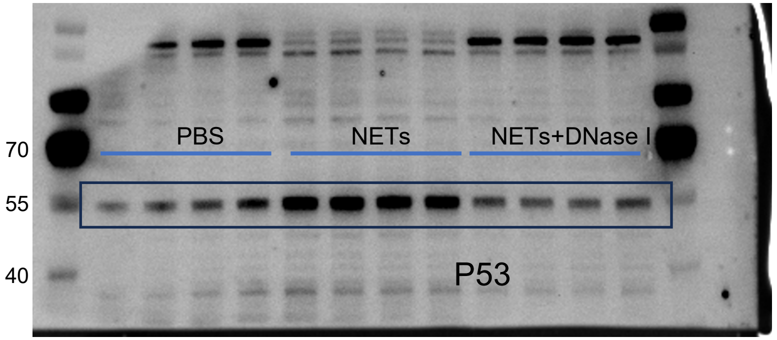

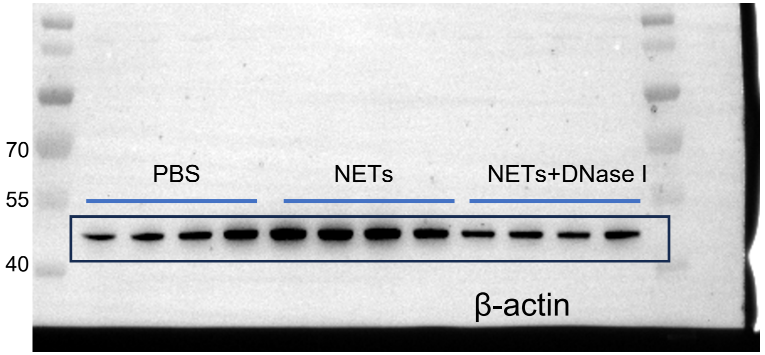

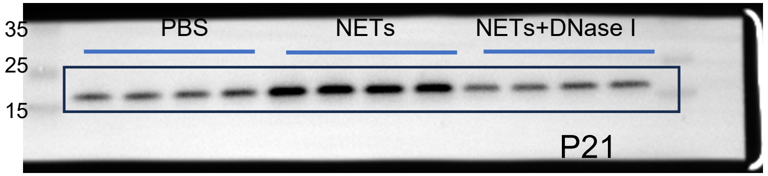

These lanes were noncontiguous on the same gel.

Full unedited blot for Supplemental Figure 4B

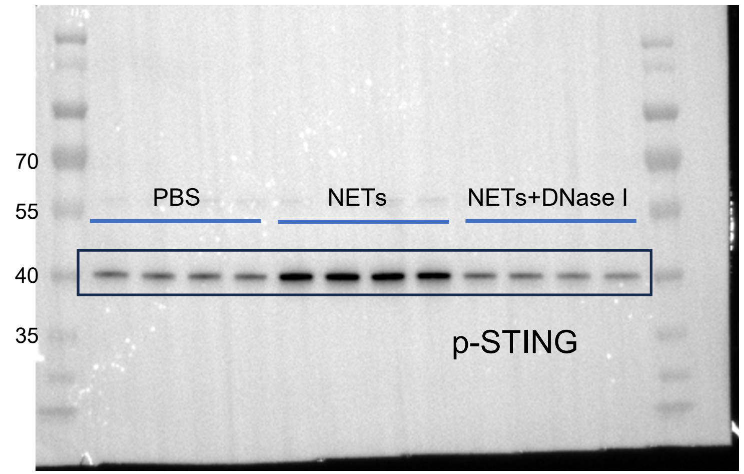

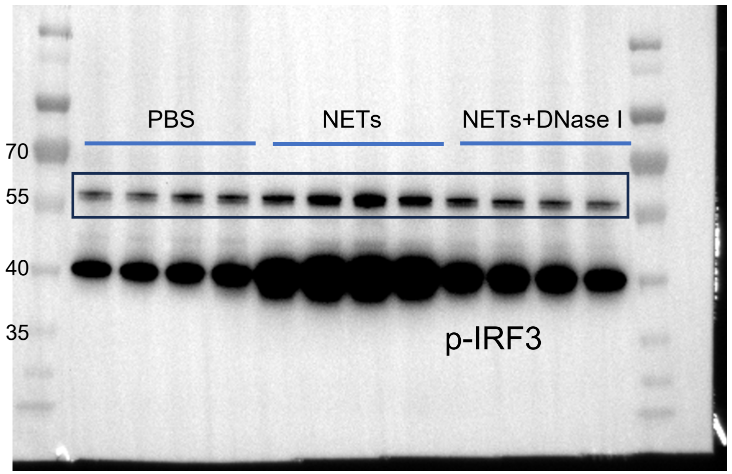

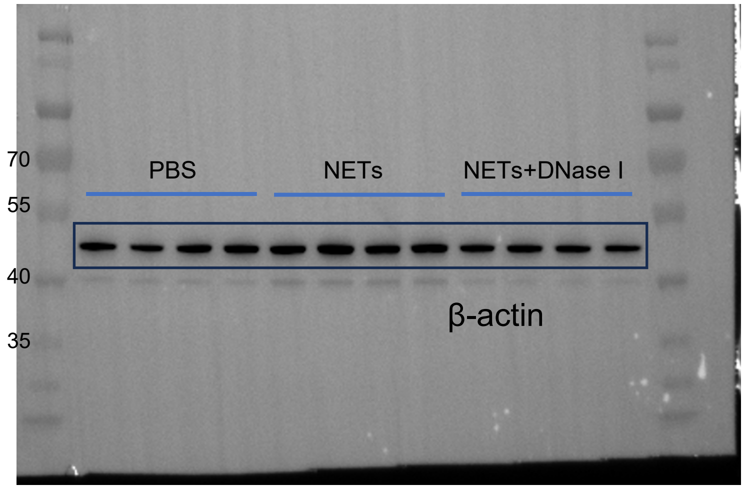

These lanes were noncontiguous on the same gel.

Full unedited blot for Supplemental Figure 4B

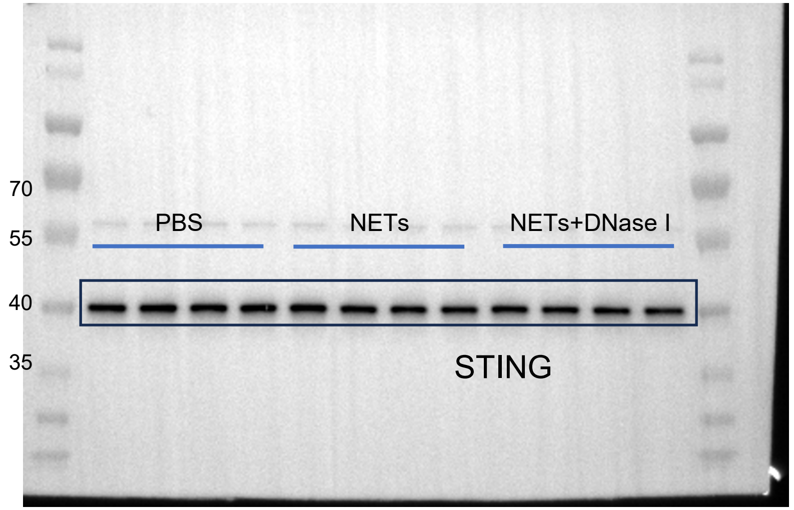

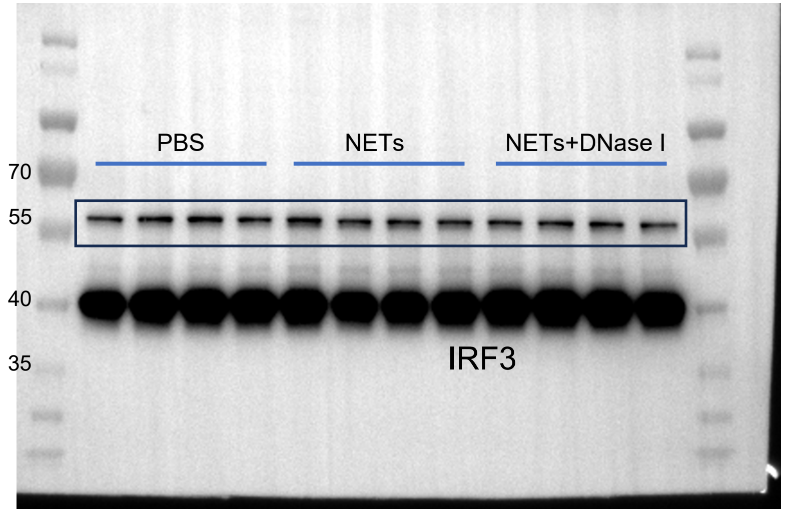

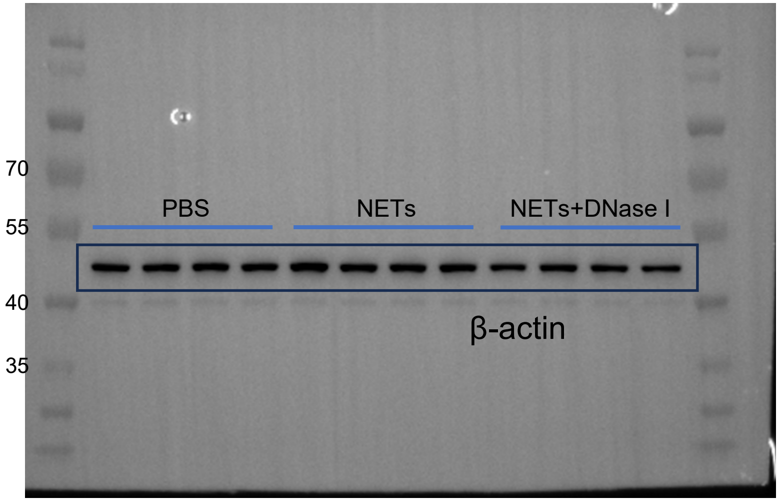

These lanes were noncontiguous on the same gel.
